# Combined Effect of Enriched Air-Cooled Blast-Furnace Slag Aggregate and Microsilica-Modified Bitumen on Asphalt Concrete Properties

**DOI:** 10.3390/ma19214598

**Published:** 2026-10-30

**Authors:** Vyacheslav Kunaev, Gulzhainat Akhmetova, Ilgar Tavshanov, Alibek Batyrbek, Dmitriy Charnyi, Bakhyt Zhautikov, Mohamad Syazli Fathi, Mohamad Zaki Hassan, Ayaulym Ospanova

**Affiliations:** 1 Department of Technological Machines and Transport, Karaganda Industrial University, Temirtau 101400, Kazakhstan; i.tavshanov@tttu.edu.kz (I.T.); a.ospanova@tttu.edu.kz (A.O.); 2 Department of Metallurgy and Material Science, Karaganda Industrial University, Temirtau 101400, Kazakhstan; g.akhmetova@tttu.edu.kz; 3 Department of Artificial Intelligence, Karaganda Industrial University, Temirtau 101400, Kazakhstan; a.batyrbek@tttu.edu.kz (A.B.); bakhyt.zhautikov@tttu.edu.kz (B.Z.); 4 Department of Science and Innovations, Karaganda Industrial University, Temirtau 101400, Kazakhstan; dep.science@tttu.edu.kz; 5 Faculty of Artificial Intelligence, Universiti Teknologi Malaysia, Kuala Lumpur 54100, Malaysia; syazli@utm.my; 6 Razak Faculty of Technology and Informatics, Universiti Teknologi Malaysia, Kuala Lumpur 54100, Malaysia; mzaki.kl@utm.my

**Keywords:** air-cooled blast-furnace slag, asphalt concrete, selective crushing, microsilica-modified bitumen, enrichment, waste recycling, Marshall stability, indirect tensile strength, moisture resistance, freeze–thaw resistance

## Abstract

**Highlights:**

- Combining enriched ACBFS aggregate and microsilica-modified bitumen improves asphalt concrete performance.
- The optimal microsilica content of 12% increases compressive strength to 5.92 MPa.
- Marshall stability reaches 20.10 kN and tensile strength 3.15 MPa with enriched ACBFS and 12% microsilica.
- SEM–EDS analysis reveals improved aggregate–mastic contact and a more continuous Si distribution.

**Abstract:**

Air-cooled blast-furnace slag (ACBFS) is a promising secondary aggregate for asphalt concrete, but its heterogeneous porosity and mechanical properties can limit performance. This study evaluates a combined approach based on selective crushing of ACBFS and modification of bitumen with microsilica. Selective crushing reduced the proportion of weak, highly porous slag particles, while microsilica was added to bitumen at 0, 3, 6, 9, 12, 15, and 18% by mass of bitumen. Asphalt concrete mixtures were assessed by density, water saturation, compressive strength, indirect tensile strength, Marshall stability and flow, water resistance, freeze–thaw resistance, and SEM–EDS analysis. The best overall performance was obtained at 12% microsilica. At this content, compressive strength reached 5.92 MPa, indirect tensile strength 3.15 MPa, Marshall stability 20.10 kN, and the water resistance coefficient 0.81, while compressive strength loss after 50 freeze–thaw cycles decreased to 8.50%. SEM observations showed fewer large interfacial voids and a more continuous aggregate–mastic contact region in the modified mixture, while EDS indicated a more continuous Si distribution. The results show that aggregate enrichment and binder modification can complement each other in improving ACBFS-based asphalt concrete.

## 1. Introduction

Achieving the United Nations Sustainable Development Goals (SDGs) requires more efficient use of natural resources, reduction in industrial waste, and development of environmentally sustainable production technologies. In particular, SDG 12 (Responsible Consumption and Production) emphasizes sustainable resource management and waste reduction through recycling and reuse. Recent studies have highlighted the importance of industrial waste recycling and its conversion into sustainable construction materials as part of the transition toward a circular economy [1,2]. Sustainable development research also addresses other complementary aspects, including the integrated utilization of geothermal resources in mining operations [3], the influence of environmental conditions on the mechanical behavior and durability of cement-based materials [4], and the environmentally safe management of metal-bearing residues through heavy-metal immobilization in cementitious matrices [5].

One of the promising approaches to supporting these sustainability objectives is the recycling of metallurgical by-products as alternative raw materials in the construction industry. Their utilization can reduce the consumption of natural mineral resources and the amount of industrial waste requiring storage. Among these materials, blast-furnace slag has attracted considerable attention because of its potential for use in various construction applications [6,7].

Depending on the cooling method, blast-furnace slag is generally classified into air-cooled blast-furnace slag (ACBFS) and granulated blast-furnace slag (GBFS). ACBFS is produced by slow cooling of molten blast-furnace slag in air, resulting in a predominantly crystalline structure. In contrast, GBFS is obtained by rapid cooling (water quenching) of molten blast-furnace slag, resulting in a predominantly amorphous (glassy) structure. Subsequent drying and grinding of GBFS produces ground granulated blast-furnace slag (GGBFS), which exhibits latent hydraulic properties and is mainly used as a supplementary cementitious material.

Unlike GGBFS, ACBFS is primarily used as a mineral aggregate in construction applications. Beyond asphalt concrete, ACBFS has been investigated as an alternative aggregate in cement-based materials. Mohamed et al. [8] examined the partial and complete replacement of natural coarse aggregate with ACBFS in conventional concrete, reporting improvements in compressive strength while identifying increased chloride permeability as a potential durability limitation at higher replacement levels. Other researchers [9] investigated the use of ACBFS as coarse aggregate in ultra-high-performance fiber-reinforced concrete, demonstrating its potential for producing construction materials with reduced consumption of natural aggregates. These studies highlight opportunities for the utilization of ACBFS in different types of cement concrete.

**Figure 1:**
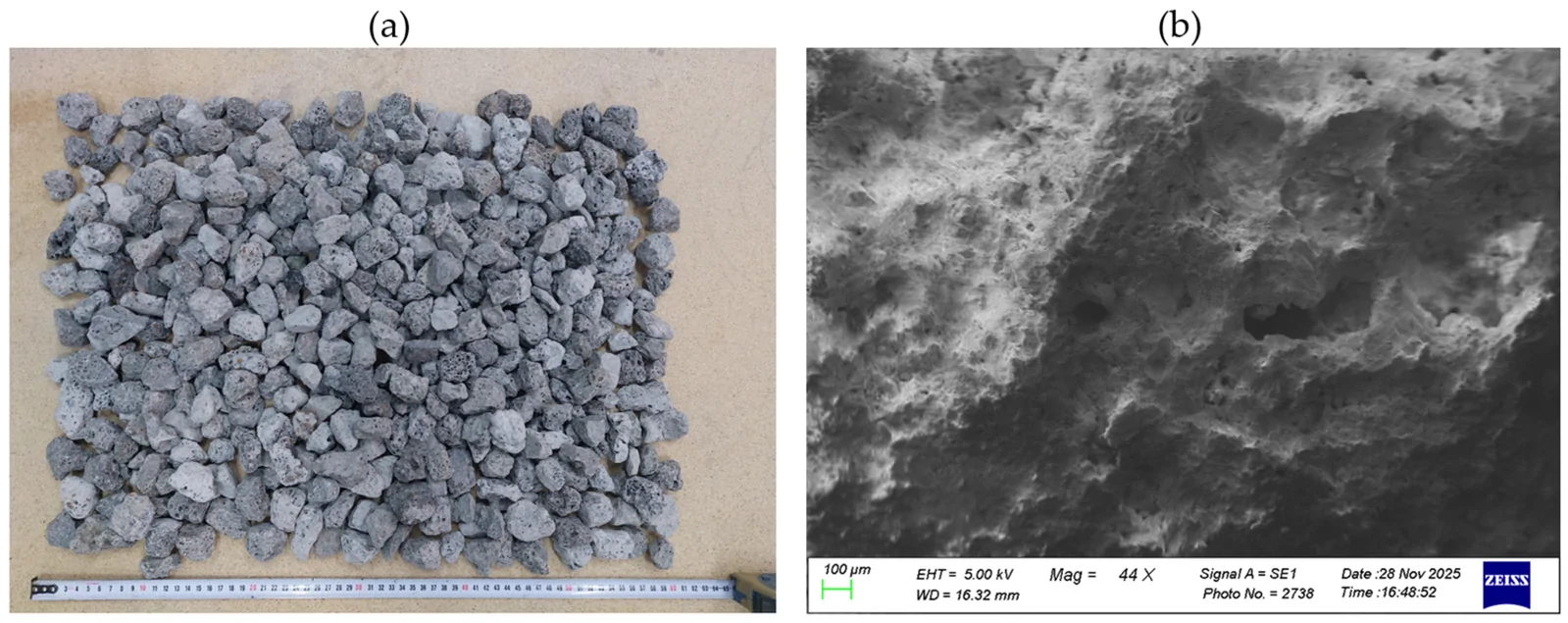
Air-cooled blast-furnace slag: (**a**) General view. (**b**) SEM micrograph.

For pavement applications, the relatively rough and porous structure of ACBFS (Figure 1) makes it a promising alternative aggregate. However, its performance in asphalt concrete depends strongly on aggregate porosity, surface characteristics, and interaction with the bituminous binder [10].

The use of ACBFS in asphalt concrete has been examined in a number of studies. Rondón-Quintana et al. investigated hot-mix asphalt in which natural coarse aggregate was replaced by blast-furnace slag and showed that mixture performance depended not only on the replacement level but also on whether the substitution was made by mass or by volume [10]. This is important because ACBFS differs considerably from conventional natural aggregate in density and internal porosity. Other studies have also shown that ACBFS affects volumetric characteristics, stiffness, moisture damage resistance, and abrasion resistance of asphalt mixtures [11]. In a recent warm-mix asphalt study, Stępień et al. found that ACBFS-containing mixtures required additional optimization compared with mixtures containing denser slag or natural aggregate, mainly because of the specific physical properties of ACBFS [12].

Surface morphology and mineral composition also influence the behavior of slag aggregate in asphalt mixtures. Moura et al. showed that the chemical composition and surface-energy characteristics of ACBFS directly affect its adhesion with bituminous binder and the moisture resistance of the resulting asphalt mixture [13]. Their results indicated that ACBFS can have weaker binder adhesion than denser steel slag, which confirms that the slag surface and internal structure should be considered when designing asphalt concrete with metallurgical aggregates.

A broader review of blast-furnace slag in pavement engineering also shows that the main limitation of ACBFS is not its suitability as a mineral material itself, but the high variability of its physical and mechanical characteristics. Kunaev et al. reported that ACBFS is mainly used as aggregate in road materials, while its porosity, water absorption, strength, and variability between individual grains remain important restrictions for wider application [14]. These limitations have led to research on preliminary processing of blast-furnace slag before its use in road materials. Kadyrov et al. proposed treating road-building blast-furnace slag aggregate by filling its pores with a cement–microsilica mixture followed by hydrophobic treatment [15]. The study showed that the excessive porosity of slag aggregate can be treated as a separate technological problem rather than only as a mixture-design issue. Other work has considered the organization of slag processing itself. Georgiadi et al. suggested a technological scheme and universal excavator equipment for preparing blast-furnace slag waste for further use in road construction. The proposed processing line was intended to produce crushed slag aggregate with improved water and frost resistance and demonstrated that slag preparation can be incorporated into a practical road-material production process [16].

Shatnawi et al. [17] showed that the use of slag aggregate can affect asphalt mixture properties, including density and Marshall stability. In their study, asphalt concrete containing 100% slag aggregate had higher density and Marshall stability than the limestone control, while the optimum bitumen content decreased from 5.3 to 4.8%. The authors associated the higher stability with the greater crushing and abrasion resistance of slag aggregate. These findings indicate that the mechanical quality of metallurgical slag aggregate is an important factor controlling asphalt concrete performance.

In study [18], a combined treatment of ACBFS aggregate, including pore filling and hydrophobic treatment, reduced water absorption from 4.54% to 1.05%. This confirms that preliminary treatment can significantly improve the water-related properties of slag aggregate before its use in road construction.

The influence of aggregate porosity on asphalt mixture performance has also been reported for other porous secondary aggregates. Zou et al. [19] showed that recycled concrete aggregate, because of its adhered porous mortar and relatively high water absorption, affected the tensile strength and moisture resistance of foamed asphalt mixtures. Al-Busaltan et al. [20] also reported that the use of recycled concrete aggregate in porous asphalt influenced moisture susceptibility, indirect tensile strength and Marshall stability. These findings indicate that the internal structure and water absorption of porous aggregate should be taken into account before its incorporation into asphalt mixtures.

Further development of the enrichment approach involved selective crushing and separation based on frictional properties. The authors of [21] showed that selective removal of weaker and more porous ACBFS particles reduced water absorption, aggregate crushing value and freeze–thaw mass loss, while hydrophobic treatment provided an additional reduction in water penetration. A similar problem has been addressed through selective crushing of coarse slag aggregate specifically intended for asphalt concrete. The process was shown to reduce water absorption from 4.96% to 2.38%, aggregate crushing value from 30.98% to 18.4%, and surface pore content from 45.06% to 15.68%, while increasing bulk density [22]. These data indicate that selective crushing changes not only the particle-size distribution but also the quality distribution of the slag aggregate by preferentially removing weak and highly porous grains.

Improvement of the aggregate itself, however, does not address all factors controlling asphalt concrete performance. Mechanical and durability properties also depend on the bituminous binder, the bitumen–mineral mastic and the aggregate–binder interface. One approach to modifying these components is the use of silica-based materials, including silica fume, microsilica and nano-silica. Several studies have shown that silica-based modifiers can influence the rheology and performance of asphalt binder. Yusoff et al. investigated nano-silica in polymer-modified asphalt and found that it reduced moisture susceptibility, increased mixture strength and improved resistance to oxidative aging [23]. Their SEM observations also showed dispersion of silica particles in the asphalt-binder matrix.

Long et al. used molecular-dynamics simulation to study the adhesion of nano-silica-modified asphalt at the aggregate interface. Their results showed that aggregate surface irregularity influences adhesion strength and that nano-silica modification can reduce sensitivity to moisture and oxidation-related degradation [24]. The effect of nano-silica on moisture susceptibility has also been confirmed experimentally. A study of nano-silica-modified asphalt concrete reported higher indirect tensile strength and improved moisture resistance compared with conventional mixtures [25]. Silica-based modification has also been investigated for porous asphalt. Masri et al. reported that the tensile-strength ratio of porous asphalt increased from 74% for the conventional mixture to 91% at an optimum nano-silica content of 2%, confirming that an appropriate silica content can significantly improve resistance to moisture-induced damage [26]. The influence of nano-SiO_2_ is not limited to moisture resistance. Chen et al. studied the combined effects of temperature, moisture and nano-SiO_2_ content on the rutting resistance of asphalt concrete and showed that the modifier affects high-temperature performance under wet conditions [27]. Silica fume has also been used in asphalt mixtures as a fine mineral component. Al-Zarjawi et al. investigated silica fume in cold-mix asphalt and reported changes in mixture performance associated with the addition of the highly dispersed silica-containing material [28].

The available literature therefore demonstrates two established but largely separate research directions. The first concerns the use and preliminary improvement of ACBFS aggregate, with particular attention to porosity, water absorption, crushing resistance and freeze–thaw durability. The second concerns modification of asphalt binder and mastic with silica-based materials to improve moisture resistance, tensile performance and rheological behavior.

Previous studies by the authors of the present paper [15,16,21,22] progressively developed methods for processing and improving blast-furnace slag aggregate, including pore treatment, technological preparation, enrichment, selective crushing, and hydrophobic modification. In contrast, independent studies on silica-modified asphalt [23,24,25,26,27,28] mainly focused on the binder and bitumen–mineral mastic system. These two research directions affect different structural components of asphalt concrete and may therefore complement each other.

The present study combines these two approaches. Regular ACBFS aggregate is first subjected to selective crushing in order to reduce the proportion of weak and highly porous grains. Separately, microsilica is introduced into the bitumen before preparation of the asphalt mixture. Thus, the coarse aggregate and the binder are modified independently before they are combined in asphalt concrete.

The aim of this study is to evaluate the combined effect of enriched ACBFS aggregate and microsilica-modified bitumen on the physical, mechanical and durability properties of asphalt concrete. Mixtures containing enriched slag aggregate and different microsilica contents are compared with control mixtures based on regular ACBFS and natural aggregate. Density, water saturation, compressive strength, indirect tensile strength, Marshall stability and flow, water resistance, and resistance to repeated freeze–thaw cycles are evaluated. SEM and EDS analyses are then used to compare the regular slag mixture with the composition that showed the best overall performance and to identify differences in aggregate morphology, the aggregate–mastic contact region and elemental distribution.

The scientific contribution of this study lies not in the individual use of selective crushing or microsilica modification, which have been investigated previously, but in the systematic evaluation of their complementary roles within a single ACBFS-based asphalt concrete system. Selective crushing improves the quality of the coarse aggregate forming the load-bearing skeleton by preferentially eliminating weak and highly porous particles, whereas microsilica modification targets the binder–mastic system and aggregate–mastic contact region. The sequential experimental design makes it possible to distinguish the improvements associated with aggregate enrichment from the additional effects of microsilica-modified bitumen and to establish how these modifications influence the physical, mechanical, and durability properties of asphalt concrete. Furthermore, the combined assessment of macroscopic performance and SEM–EDS observations provides insight into the structural changes associated with the observed improvements. Thus, the novelty of the present work lies in the evaluation of a two-level modification strategy that combines distinct mechanisms of material improvement, each addressing different factors governing the physical, mechanical, and durability properties of slag-based asphalt concrete.

## 2. Materials and Methods

### 2.1. Experimental Design

**Figure 2:**
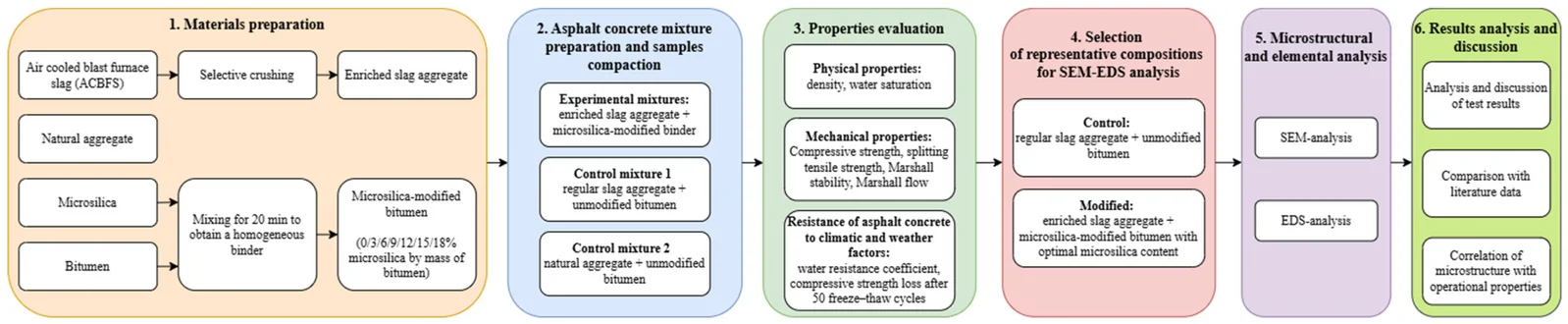
Experimental workflow.

The experimental program was designed to evaluate the combined effect of preliminary enrichment of air-cooled blast-furnace slag (ACBFS) aggregate and modification of bitumen with microsilica on the properties of asphalt concrete. The experimental workflow is presented in Figure 2.

The experimental design was based on the successive improvement of asphalt concrete components. Initially, the effectiveness of selective crushing was evaluated by comparing regular and enriched ACBFS aggregates and the corresponding asphalt concrete mixtures prepared with unmodified bitumen. Subsequently, the enriched ACBFS aggregate was used to investigate the additional effect of microsilica modification of the bituminous binder on asphalt concrete properties. Different microsilica contents were examined to determine the optimal dosage for asphalt concrete containing enriched slag aggregate. Thus, the experimental program focused on evaluating the successive improvements in material properties rather than quantifying the synergistic interaction between aggregate enrichment and binder modification.

The study consisted of six main stages. First, the initial materials were prepared, including regular ACBFS, natural aggregate, bitumen, and microsilica. Regular ACBFS was subjected to selective crushing to obtain enriched slag aggregate with a reduced proportion of weak and high-porosity grains. In parallel, bitumen was modified with microsilica added at 0, 3, 6, 9, 12, 15, and 18% by mass of bitumen. The prepared aggregate and binder were then used to produce experimental asphalt concrete mixtures and two control mixtures. The specimens were evaluated in terms of physical, mechanical, water-related, and freeze–thaw properties. Based on the overall test results, two representative compositions were selected for subsequent SEM and EDS analyses. Finally, the microstructural observations were compared with the measured performance characteristics and discussed in relation to published data.

### 2.2. Aggregate Preparation

**Figure 3:**
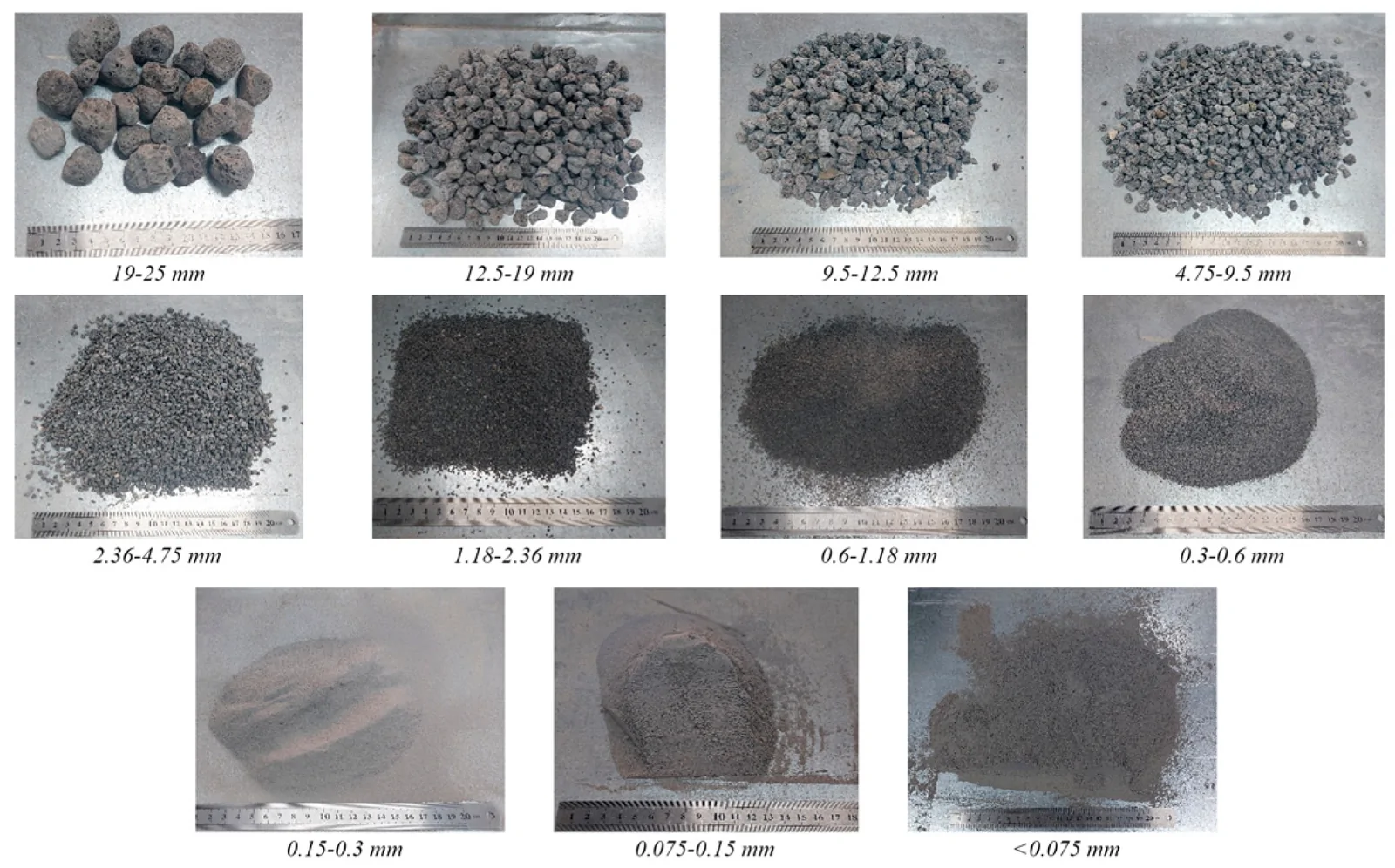
Particle-size fractions of blast-furnace slag aggregate used for preparation of the asphalt concrete mixture.

**Figure 4:**
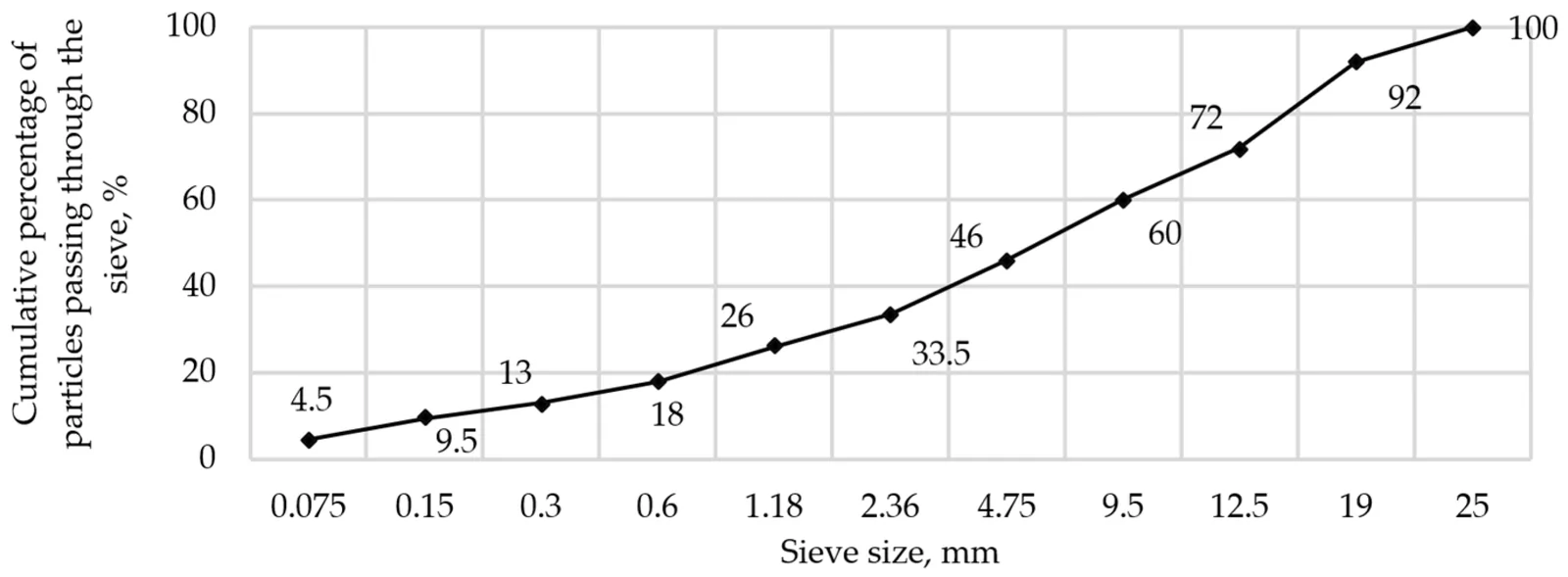
Particle-size distribution curve of the aggregate used in the experimental asphalt concrete mixtures.

ACBFS was used as the main mineral slag aggregate for preparation of the experimental asphalt concrete mixtures. Before mixture preparation, the slag aggregate was separated into individual particle-size fractions. The fractions subsequently used to form the aggregate grading of the asphalt concrete are shown in Figure 3, while the cumulative particle-size distribution of the final aggregate blend is presented in Figure 4.

Chemical composition of ACBFS is presented in Table 1. The slag is mainly composed of CaO and SiO_2_, with contents of 44.3 and 40.7 wt.%, respectively, while Al_2_O_3_ and MgO are present in lower amounts. The predominance of Ca- and Si-containing components is typical of blast-furnace slag and is consistent with the elemental signals later observed by EDS analysis. The relatively high contents of CaO and SiO_2_ also indicate that the mineral phase of the slag is substantially different from that of natural aggregate, which should be taken into account when interpreting the microstructural and interfacial characteristics of the asphalt concrete.

Regular ACBFS is structurally heterogeneous and contains grains that differ considerably in density, porosity, and mechanical strength. Highly porous particles are generally weaker and more susceptible to fragmentation, whereas denser particles exhibit higher mechanical resistance. To reduce the proportion of weak and highly porous grains, the coarse ACBFS fraction was subjected to preliminary enrichment by selective crushing.

**Figure 5:**
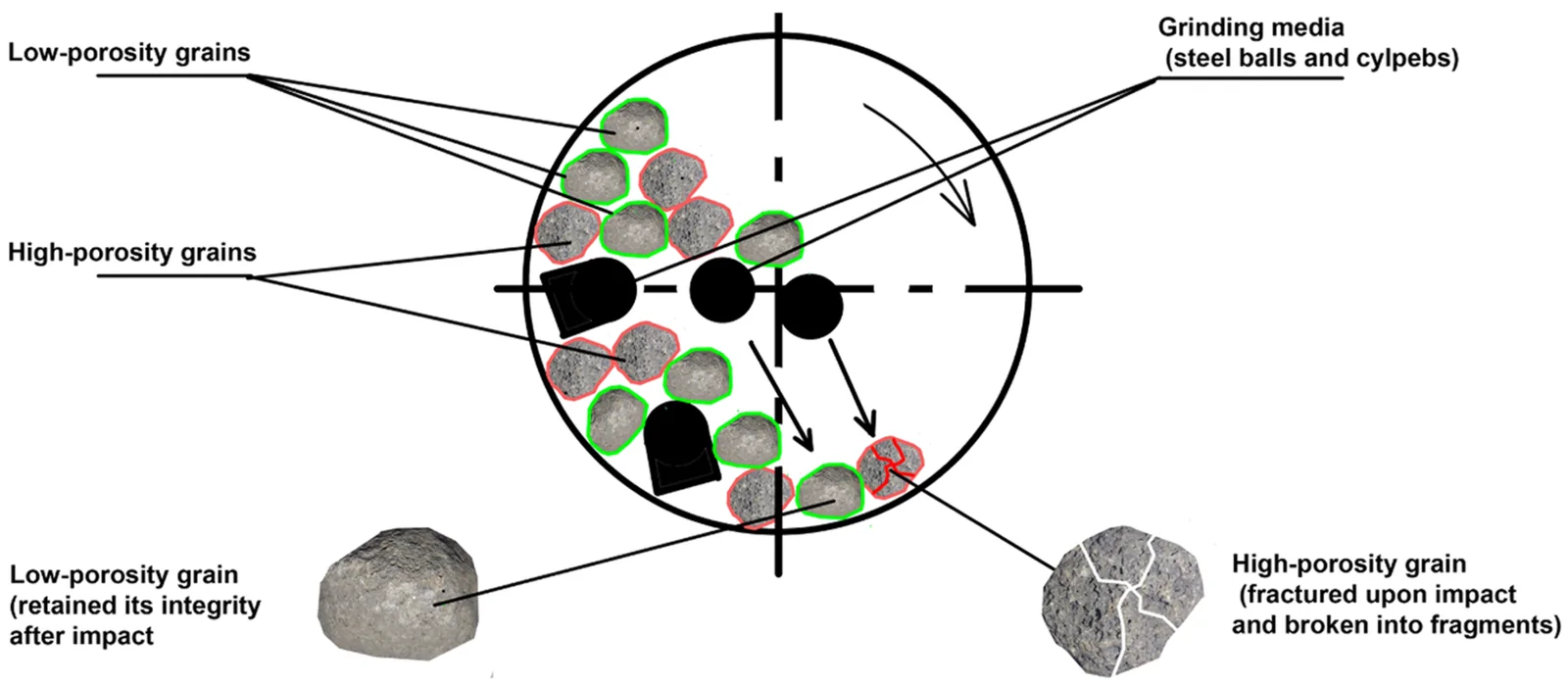
Schematic representation of the selective crushing mechanism used for enrichment of blast furnace slag aggregate.

Selective crushing was carried out in a laboratory ball mill. The mechanism of selective crushing is illustrated schematically in Figure 5. During rotation of the mill chamber, the slag particles interacted with the grinding media, which consisted of 50% steel balls and 50% steel cylpebs, with a diameter of 100 mm. This combination had previously been found [15] to provide the most selective crushing action: highly porous and mechanically weak grains were preferentially fractured, while denser and stronger grains largely retained their integrity. This difference in response to mechanical loading formed the basis of the enrichment process. The combined grinding media therefore provided a balance between effective destruction of low-quality grains and preservation of structurally sound aggregate particles [15]. Selective crushing was performed for 40 min.

After 40 min of selective crushing, the initially prepared 20–25 mm ACBFS fraction was sieved through a 20 mm sieve to separate the particles that retained their original integrity from those fragmented during milling. The retained fraction, consisting predominantly of dense, low-porosity, and mechanically resistant particles, accounted for approximately 35% of the initial slag volume, as established in our previous study [22]. These enriched particles were used as the 19–25 mm coarse aggregate fraction, forming the primary load-bearing skeleton of the asphalt concrete mixture. Importantly, the remaining approximately 65% of the slag was not discarded. Instead, the fragmented particles were further crushed and sieved into smaller fractions (2.36–4.75 mm, 1.18–2.36 mm, 0.6–1.18 mm, etc.), which were also incorporated into the asphalt concrete mixture as non-enriched slag aggregate. Thus, both the coarse and fine aggregate fractions consisted entirely of ACBFS, while selective enrichment was applied specifically to the largest particles responsible for forming the load-bearing aggregate skeleton.

The primary purpose of selective crushing was to ensure that weak and highly porous coarse slag particles were fractured during the preliminary crushing stage rather than at later stages of pavement construction or operation. In particular, the fracture of such particles during roller compaction could expose fresh aggregate surfaces (after splitting the grains) that are not coated with bitumen, potentially weakening the adhesion between the aggregate and the bituminous binder. Alternatively, their fracture under repeated heavy traffic loads during pavement service could disrupt the load-bearing aggregate skeleton and lead to localized settlement and deformation of the asphalt pavement. Thus, selective crushing allows mechanically weak particles to be broken down in advance, ensuring that only the strongest and least porous coarse slag particles are retained to form the primary load-bearing skeleton of the asphalt concrete. The particle-size distribution of the final aggregate blend is presented in Figure 4.

The effect of enrichment was evaluated by comparing the physical and mechanical properties of regular ACBFS, enriched ACBFS, and natural aggregate (Table 2). Compared with regular ACBFS, the enriched slag aggregate had a higher bulk density and substantially lower water absorption, abrasion loss, content of flaky and elongated particles, and aggregate crushing value. Bulk density increased from 1197 to 1390 kg/m^3^, while water absorption decreased from 4.98 to 2.38%. The content of flaky and elongated grains decreased from 12.42 to 0.24%, abrasion loss decreased from 45.54 to 32.05%, and the aggregate crushing value decreased from 30.82 to 18.4%.

These changes indicate that selective crushing modified not only the particle-size distribution but also the quality distribution of the slag aggregate. By preferentially removing weak and highly porous particles, the enrichment procedure increased the relative proportion of dense and mechanically stable ACBFS grains used in the asphalt concrete mixtures.

### 2.3. Bitumen and Microsilica

**Figure 6:**
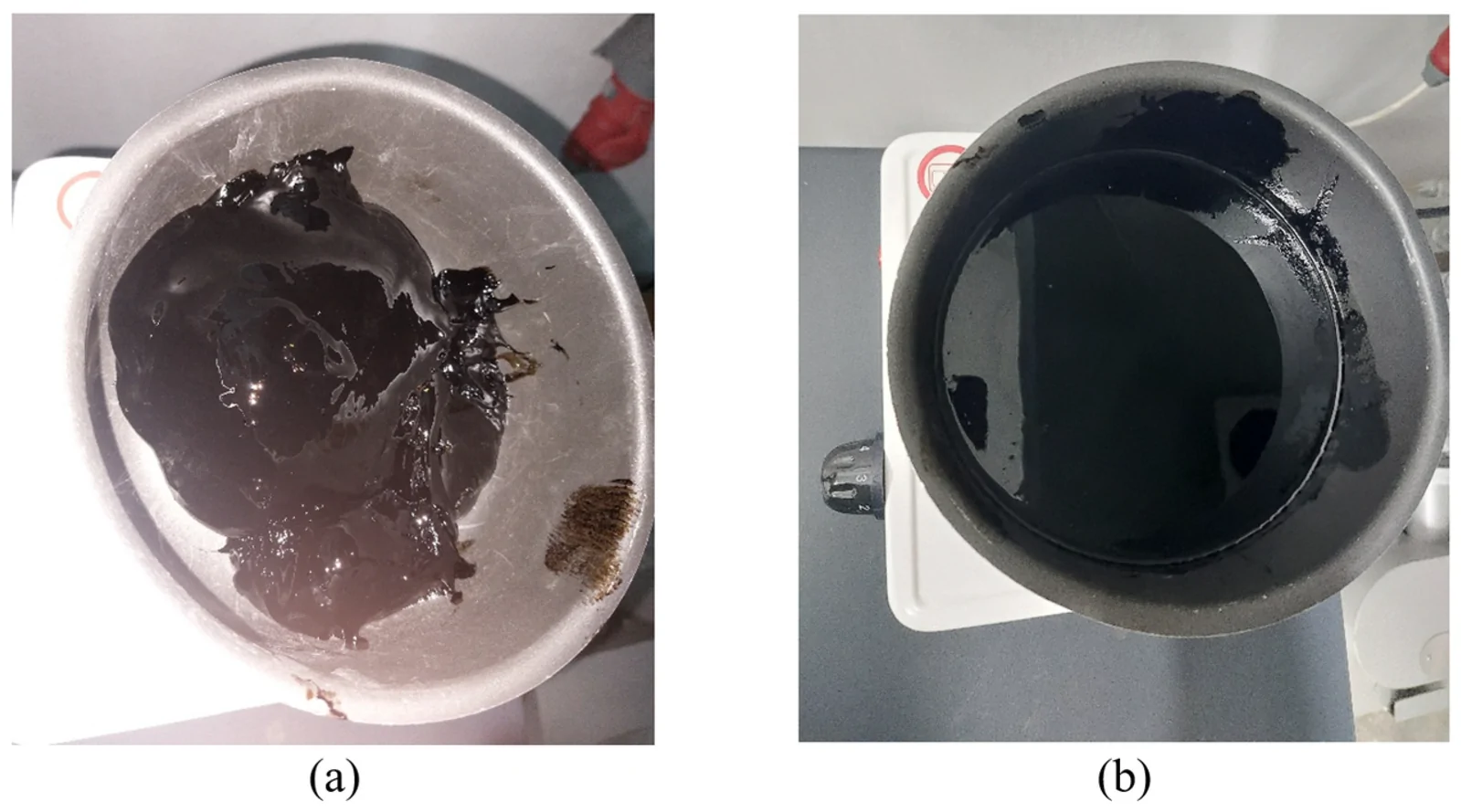
Bituminous binder used for preparation of the experimental asphalt concrete mixtures: (**a**) at room temperature; (**b**) in the heated fluid state.

The bituminous binder used for preparation of the asphalt concrete mixtures was paving bitumen BND 100/130 produced by JSC KazBitumService. The main characteristics of the bituminous binder are summarized in Table 3. The binder is shown in Figure 6 at room temperature and in the heated fluid state. Before modification and mixing with the mineral aggregate, the bitumen was heated to approximately 140 °C. The bituminous binder content in the asphalt mixture was 5% by mass of the mineral component.

**Figure 7:**
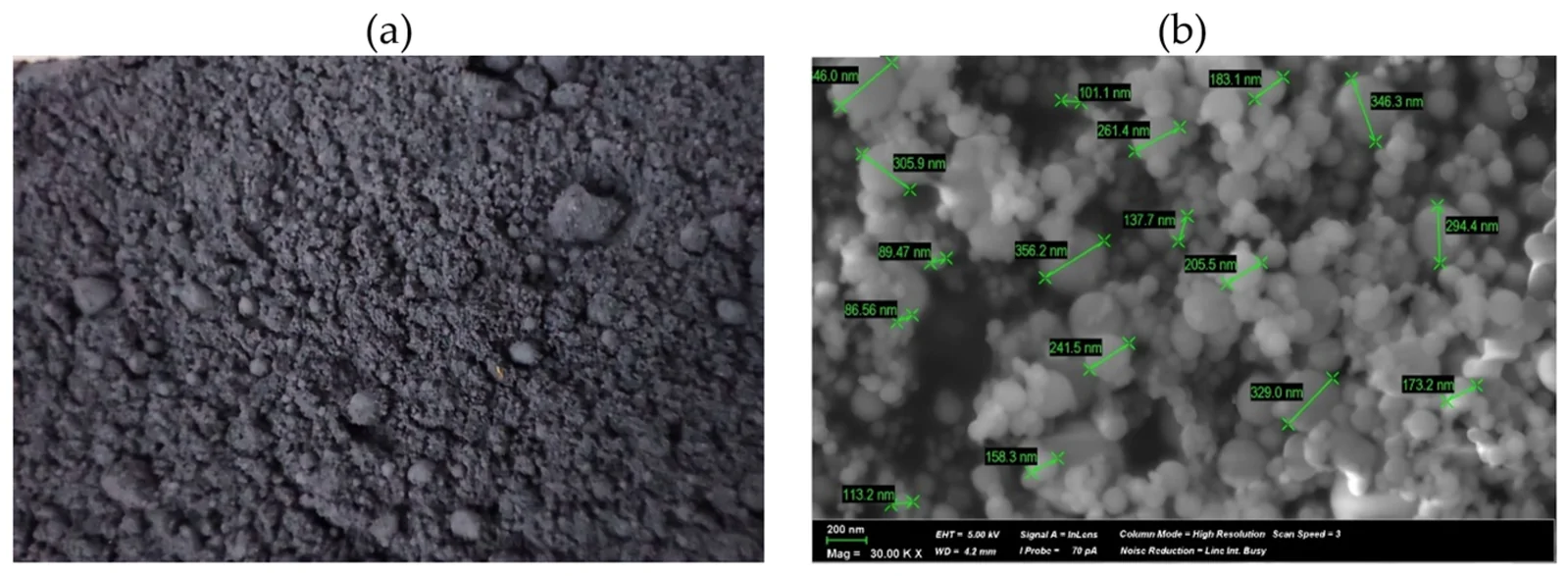
Microsilica: (**a**) general view; (**b**) SEM micrograph (adapted from [38]).

Undensified microsilica produced by YDD (Karaganda, Kazakhstan) was used as the modifier. Its bulk density was 340 kg/m^3^. The material contained approximately 94.37 wt.% SiO_2_; the remaining chemical components are given in Table 4. The general appearance and SEM morphology of the microsilica are shown in Figure 7 [38].

Microsilica was introduced directly into the heated bitumen at contents of 0, 3, 6, 9, 12, 15, and 18% by mass of bitumen. After addition of the required amount of microsilica, the binder was mixed for 20 min until a homogeneous state was obtained.

### 2.4. Preparation of Asphalt Concrete Mixtures and Specimens

**Figure 8:**
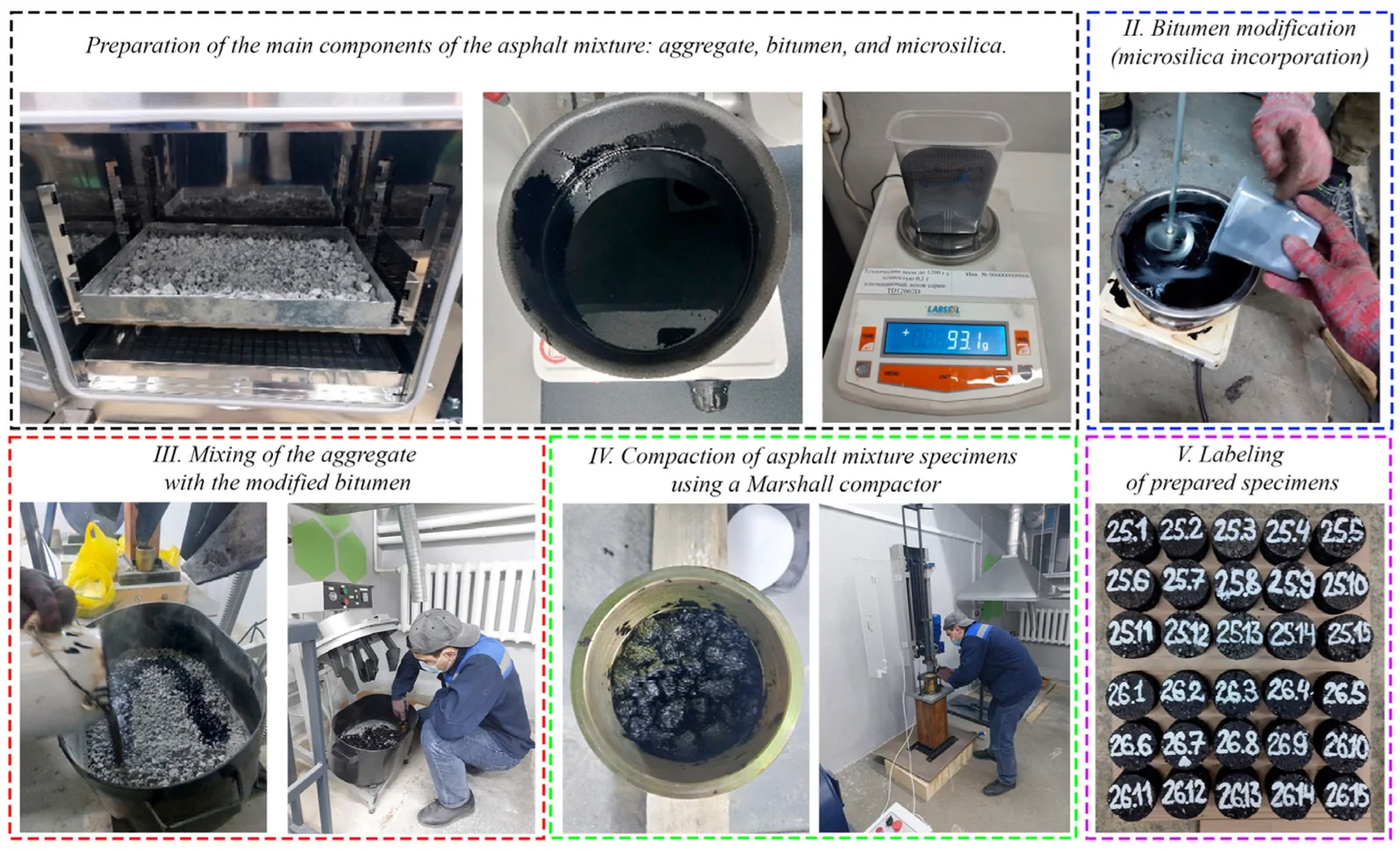
Experimental procedure for preparation of experimental asphalt concrete specimens using microsilica-modified bitumen.

The procedure used for preparation of the asphalt concrete specimens is shown in Figure 8. The aggregate fractions, bitumen, and microsilica were prepared separately before mixing. The required quantity of microsilica was first incorporated into the heated bitumen and mixed for 20 min until a homogeneous microsilica-modified binder was obtained.

The modified binder was subsequently mixed with the prepared mineral aggregate. The resulting asphalt mixture was placed in cylindrical Marshall molds and compacted using a Marshall compactor. Each specimen was compacted by 50 blows on each side. After compaction and cooling, the specimens were removed from the molds, labelled according to mixture composition, and used for subsequent physical, mechanical, water-resistance, and freeze–thaw tests.

The main experimental series consisted of asphalt concrete containing enriched ACBFS aggregate and bitumen with 0, 3, 6, 9, 12, 15, or 18% microsilica. Two additional control mixtures were prepared for comparison:

Control mixture 1 contained regular, non-enriched slag aggregate and unmodified bitumen.

Control mixture 2 contained natural aggregate and unmodified bitumen.

This experimental design made it possible to distinguish between the effect associated with enrichment of the slag aggregate and the additional effect associated with microsilica modification of the binder.

### 2.5. Determination of Physical Properties

**Figure 9:**
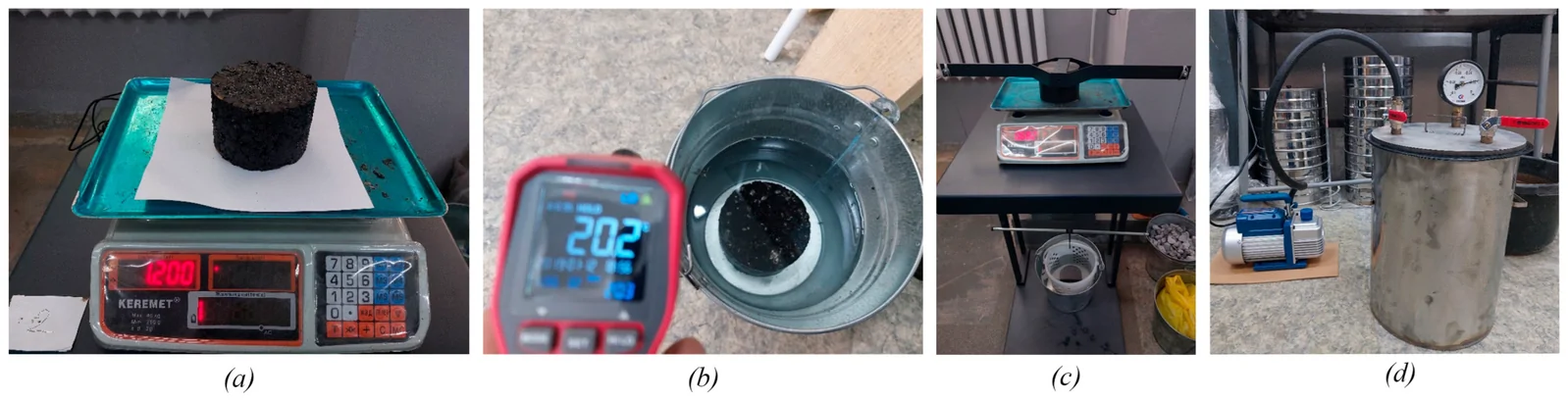
Experimental procedure for determining the physical properties of experimental asphalt concrete specimens: (**a**) measuring specimen mass in air; (**b**) conditioning in a water reservoir at 20 ± 2 °C; (**c**) determination of submerged mass; (**d**) vacuum saturation prior.

The physical properties of the asphalt concrete specimens were evaluated in terms of average density and water saturation in accordance with [39]. The main stages of the procedures are illustrated in Figure 9.

Average density was determined by hydrostatic weighing. Each specimen was first weighed in air, then immersed in water at 20 ± 2 °C for 30 min, with the water level maintained at least 20 mm above the specimen surface. The specimen was subsequently weighed while submerged in water, ensuring that no air bubbles remained on its surface. After removal from the water, the specimen was wiped with a soft cloth and weighed again in air. The average density, ρ, was calculated as:

ρ = gρ_w_/(g_2_ − g_1_). [g/cm^3^] (1)

where g is the mass of the dry specimen in air (g), g_1_ is the mass of the specimen in water (g), g_2_ is the mass of the specimen after 30 min of water conditioning and subsequent weighing in air (g), and ρ_w_ is the density of water, taken as 1 g/cm^3^.

Water saturation was determined using specimens for which the density had already been measured. The specimens were placed in water at 20 ± 2 °C, with the water level at least 30 mm above their surface, and subjected to vacuum saturation at a pressure not exceeding 2000 Pa for 1 h. After restoring atmospheric pressure, the specimens were kept in the same water bath for an additional 30 min. They were then weighed in the saturated condition.

Water saturation, W, was calculated as:

W = (g_5_ − g)100/(g_2_ − g_1_). [%] (2)

where g_5_ is the mass of the water-saturated specimen in air (g), and the remaining symbols are as defined above.

### 2.6. Determination of Mechanical Properties

**Figure 10:**
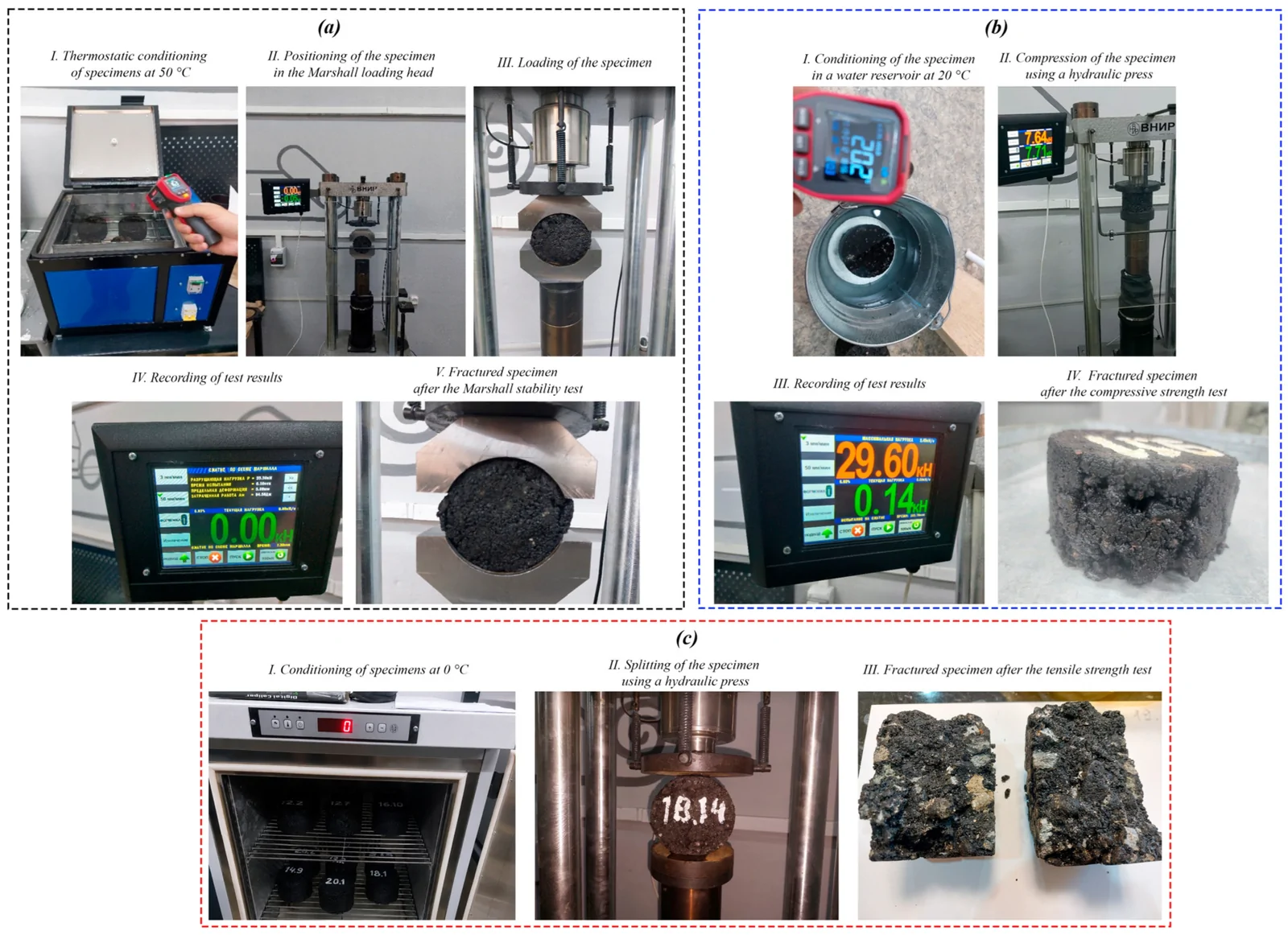
Experimental procedures for evaluating the mechanical properties of asphalt concrete specimens: (**a**) Marshall stability test; (**b**) compressive strength test; and (**c**) ITS test.

The mechanical properties of the asphalt concrete were evaluated in terms of Marshall stability and flow, compressive strength, and indirect tensile strength (ITS) in accordance with GOST 12801-98 [39]. Three specimens were tested for each mixture composition. The principal test procedures are shown in Figure 10.

For the Marshall test, the specimens and loading head were conditioned in water at 50 ± 2 °C for 1 h. The specimens were then tested using an IP-1A-500 AB PK “Universal” asphalt testing machine equipped with a Marshall loading head. The loading rate was 50.0 ± 1.0 mm/min. The maximum recorded load was taken as the Marshall stability (kN), while the corresponding deformation was recorded as Marshall flow (mm).

Compressive strength was determined on Marshall-compacted specimens after conditioning in water at 20 ± 2 °C. The specimens were loaded axially using the same testing machine at a platen displacement rate of 3.0 ± 0.3 mm/min until failure. The compressive strength, R_c_ (MPa), was calculated as:

R_c_ = P·10^−2^/F [MPa] (3)

where P is the failure load (N) and F is the initial cross-sectional area of the specimen (cm^2^).

ITS was determined by diametral compression of cylindrical specimens. Before testing, the specimens were conditioned at 0 °C. Each specimen was then positioned centrally between the loading platens and loaded along its diameter at a constant platen displacement rate of 3.0 ± 0.3 mm/min until splitting failure occurred. The maximum recorded load was taken as the failure load. According to GOST 12801-98, the indirect tensile strength, R_ITS_, was calculated as:

R_ITS_ = P·10^−2^/hd [MPa] (4)

where P is the failure load (N), h is the specimen height (cm), and d is the specimen diameter (cm).

### 2.7. Evaluation of the Asphalt Concrete Resistance to Climatic and Weather Factors

**Figure 11:**
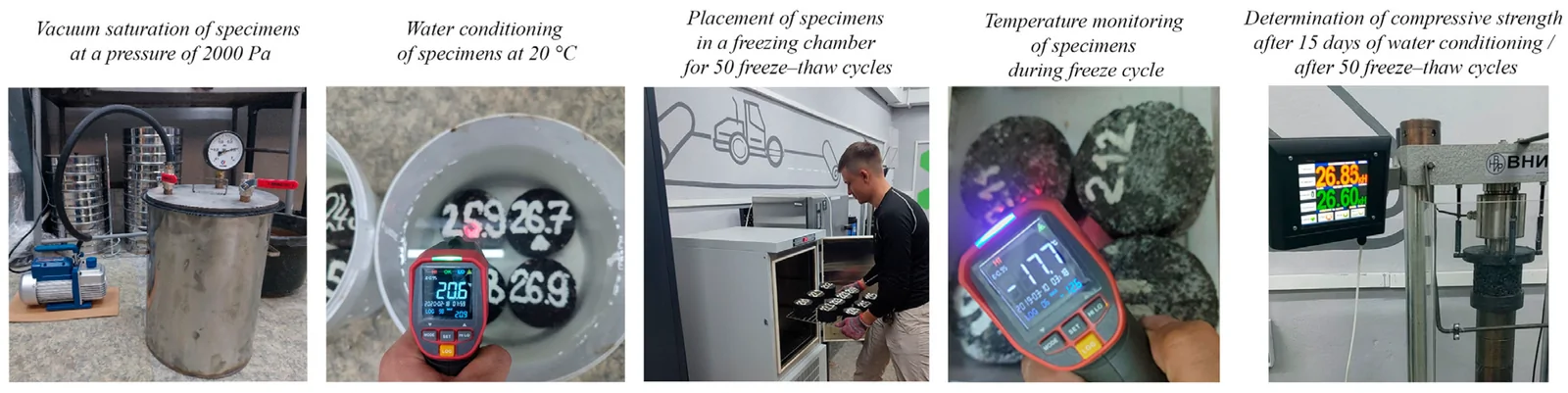
Experimental procedure for determining the water resistance coefficient and freeze–thaw resistance of asphalt concrete specimens.

The resistance of the asphalt concrete specimens to climatic and weather factors was evaluated in terms of the water resistance coefficient after prolonged water conditioning and compressive strength loss after 50 freeze–thaw cycles in accordance with [39]. The main stages of the procedures are illustrated in Figure 11.

For determination of water resistance, the specimens were first vacuum-saturated with water and then stored in water at 20 °C for 15 days. After conditioning, the specimens were removed from the water, surface-dried, and tested for compressive strength. The water resistance coefficient, K_w_, was calculated as the ratio of the compressive strength after 15 days of water conditioning to the compressive strength of the corresponding specimens tested without prolonged water saturation:

K_w_ = R_c,w15_/R_c,0_ (5)

where R_c,w15_ is the compressive strength after 15 days of water conditioning (MPa), and R_c,0_ is the compressive strength of the reference specimens (MPa).

Freeze–thaw resistance was determined using water-saturated specimens subjected to 50 freeze–thaw cycles. During each cycle, the specimens were frozen in a climatic chamber at −18 °C and subsequently thawed in water. The specimen temperature was monitored during cycling. After completion of 50 cycles, the specimens were conditioned in water and their compressive strength was determined.

The compressive strength loss after freeze–thaw cycling, ΔR_c loss_ (%), was calculated as:

ΔR_c loss_ = 100(R_c,w_ − R_c,FT_)/R_c,w_ [%] (6)

where R_c,w_ is the compressive strength of water-saturated specimens before freeze–thaw cycling (MPa), and R_c,FT_ is the compressive strength after 50 freeze–thaw cycles (MPa). A lower value of ΔR_c loss_ indicates higher freeze–thaw resistance.

### 2.8. SEM and EDS Analysis

SEM and EDS examinations were not performed for every microsilica dosage. Instead, two representative mixtures were selected after completion of the physical, mechanical, and durability tests.

The first was Control mixture 1, containing regular aggregate and unmodified bitumen. This mixture represented the initial slag asphalt concrete without either aggregate enrichment or binder modification.

The second was the mixture containing enriched slag aggregate and 12% microsilica-modified bitumen. This composition was selected because the preceding tests showed that it provided the best overall combination of the investigated characteristics. In the Results and Discussion section, this mixture showed the maximum or near-maximum values of density, compressive strength, ITS, Marshall stability, and water resistance, together with the lowest compressive strength loss after 50 freeze–thaw cycles.

Selection of these two compositions therefore allowed the microstructure of the initial slag asphalt concrete to be compared directly with that of the composition showing the strongest overall effect of the combined aggregate and binder modification.

**Figure 12:**
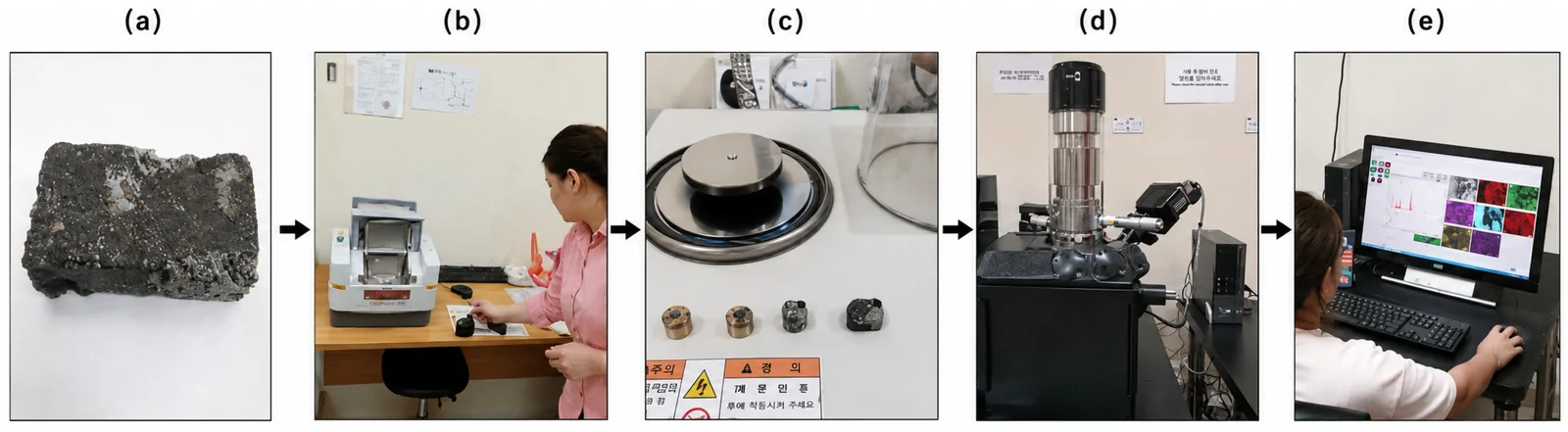
Workflow of SEM–EDS analysis of experimental asphalt concrete specimens: (**a**) cut asphalt fragment; (**b**) sample preparation; (**c**) samples mounted on SEM stubs; (**d**) scanning electron microscope (SEM) system; (**e**) energy-dispersive X-ray spectroscopy (EDS) mapping (The Korean inscriptions visible in the photographs are original equipment labels and safety warnings).

The sequence of cutting, sample preparation, mounting, SEM examination, and EDS mapping is shown in Figure 12. SEM and EDS analyses were performed using a JEOL IT300(LA) scanning electron microscope (JEOL Ltd., Japan) equipped with an energy-dispersive X-ray spectroscopy system. Fragments were cut from the selected asphalt concrete specimens, prepared for microscopic examination, mounted on SEM stubs, and examined at several magnifications.

SEM observations were used to compare the morphology of the regular and enriched slag particles, the occurrence of pores and interparticle voids, and the structure of the contact region between the slag aggregate and the surrounding bitumen–mineral mastic.

EDS mapping was used to examine the spatial distribution of the principal detected elements, particularly Si, Ca, and Al. The control and modified specimens were analyzed at the same magnification to allow qualitative comparison of elemental distribution.

In addition, representative EDS spectra were selected and compared for the control and modified mixtures. For each composition, a spectrum corresponding most closely to the average elemental composition of the examined fields with respect to the principal mineral elements Mg, Al, Si, and Ca was selected. This approach was used to illustrate a characteristic elemental profile without treating a single local spectrum as representative of the entire heterogeneous asphalt concrete specimen.

### 2.9. Analysis and Interpretation of Experimental Results

The experimental results were analyzed in three stages. First, the effect of microsilica content and aggregate type on the measured asphalt concrete properties was evaluated by comparing the experimental mixtures with both control compositions. Particular attention was given to the existence of an intermediate microsilica content providing the best overall combination of properties.

Second, the obtained trends were compared with previously published results for slag-containing and silica-modified asphalt mixtures.

Finally, the SEM and EDS observations were considered together with the macroscopic test results in order to establish a qualitative correlation between microstructure and operational properties. The analysis focused on differences in slag particle porosity, the continuity of the aggregate–mastic contact region, the occurrence of interfacial voids, and the distribution of the main elements between the control and selected modified mixtures.

## 3. Results and Discussion

### 3.1. Effect of Combined Use of Enriched Slag Aggregate and Microsilica-Modified Bitumen on the Physical Properties of Asphalt Concrete

**Figure 13:**
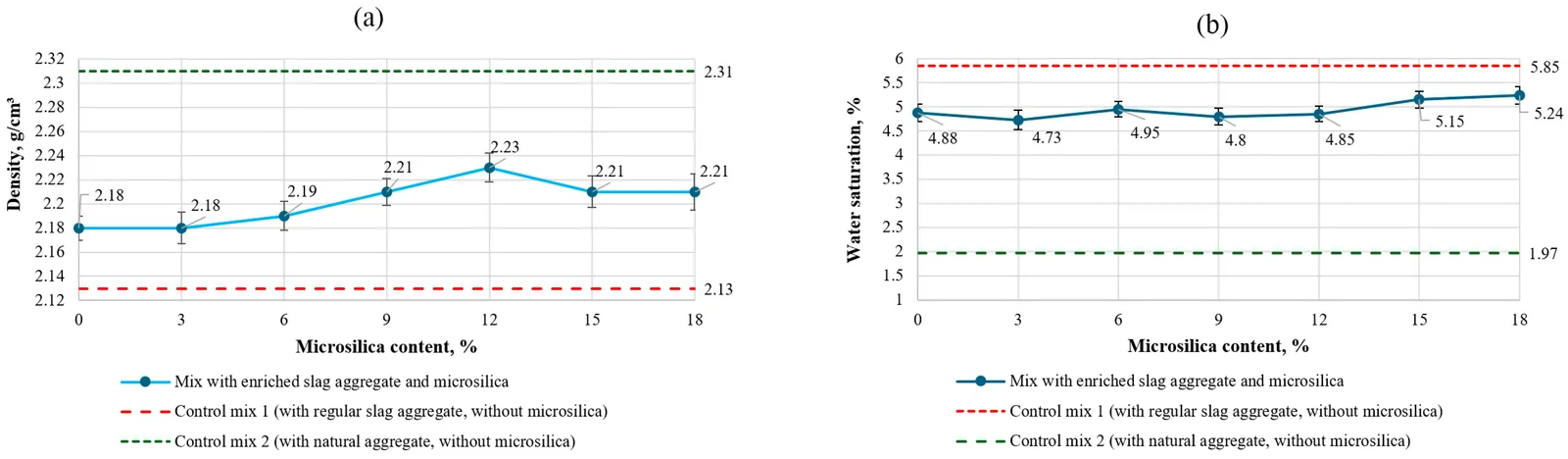
Effect of microsilica content on the physical properties of asphalt concrete with enriched slag aggregate compared with control mixtures: (**a**) density; (**b**) water saturation.

The results of evaluating the effects of slag aggregate enrichment and bitumen modification with microsilica on the physical properties (density and water saturation) of asphalt concrete are shown in Figure 13. The control mixtures with natural aggregate and regular slag aggregate are shown for comparison.

As can be seen from Figure 13a, the density changed within a relatively narrow range. For mixtures with enriched slag aggregate, it increased from about 2.18 g/cm^3^ without microsilica to a maximum of about 2.23 g/cm^3^ at 12% microsilica and then decreased slightly at 15 and 18%. Water saturation remained relatively stable up to 12% microsilica and increased at higher contents (Figure 13b). The control mixture with natural aggregate had a higher density (2.31 g/cm^3^) and, accordingly, lower water saturation (1.97%) than all mixtures containing ACBFS and microsilica. This can be attributed to the denser and less porous structure of the natural aggregate. Even after enrichment, ACBFS retains part of its characteristic intragranular porosity formed during slag cooling, which contributes to higher water absorption and a lower overall mixture density. At the same time, the mixtures with enriched slag aggregate generally showed lower water saturation (4.73–5.24%) than the control mixture with regular slag aggregate (5.85%).

The difference between the regular and enriched slag mixtures should first be related to the properties of the coarse aggregate. ACBFS is heterogeneous and may contain both dense and highly porous grains. Selective crushing can remove part of the weak and porous fraction. In a previous study of ACBFS enrichment by selective crushing, bulk density increased from 1169 to 1396 kg/m^3^, water absorption decreased from 4.96 to 2.38%, and aggregate crushing value decreased from 30.98 to 18.4%. Surface pore content also decreased from 45.06 to 15.68% [22]. Similar effects were reported for slag enrichment by grain density and friction properties, where removal of weaker porous particles reduced water absorption and improved crushing and freeze–thaw resistance [21].

These data support the difference observed in the present study between mixtures containing regular and enriched slag aggregate. The lower water saturation of the enriched-slag mixtures can therefore be partly explained by the lower proportion of highly porous coarse particles.

Despite the improvements achieved through selective crushing, enriched ACBFS still exhibits higher water absorption (2.38%) and abrasion loss (32.05%) than natural aggregate (0.52% and 11.15%, respectively; Table 2). Nevertheless, these characteristics do not necessarily preclude its use in asphalt pavements, particularly on low-load roads. In Kazakhstan, standards ST RK 1376-2005 [40] and GOST 32826-2014 [41] regulate the use of slag aggregates in road construction. According to the Russian standard GOST R 58401.1-2019 [42], aggregates with a crushing and wear resistance grade of at least I5 (corresponding abrasion loss 30–40%) are recommended for asphalt surface courses under light traffic conditions. The investigated aggregate, with approximately 32% mass loss, may satisfy this requirement. International specifications also recognize differentiated requirements for slag aggregates. The Australian MRTS101 (2022) [43] establishes a maximum water absorption of 2.5% for coarse aggregates, slightly above the 2.38% obtained in this study. The US INDOT guidelines (2013, Chapter 5) [44] specify a maximum absorption of 5% for certain aggregate classes and explicitly exempt blast furnace slag from abrasion requirements. Similarly, the British MCHW Series 900 (2021, Clause 901) specifies LA50 for blast furnace slag compared with LA30 for natural aggregates and excludes blast furnace slag from the specified water absorption requirements [45]. Furthermore, EN 13043:2002 excludes water absorption testing as a screening criterion for the freeze–thaw resistance of blast furnace slag [46]. These provisions support the potential use of enriched ACBFS in selected asphalt pavement applications, subject to verification of the applicable material and mixture requirements.

Microsilica provides an additional effect after aggregate enrichment. The increase in density up to 12% microsilica is small, but it is consistent with the introduction of very fine silica particles into the bitumen and the resulting bitumen–mineral mastic. The fine particles can occupy part of the small spaces between larger mineral components. Similar filling effects have been reported for silica-fume-modified asphalt mixtures. In a study [47] where silica fume was used as mineral filler, the modified mixtures showed mechanical and volumetric properties comparable to or slightly better than mixtures containing conventional filler.

The increase in water saturation at 15 and 18% microsilica shows that a higher modifier content is not always beneficial. A larger amount of fine silica increases the total surface area of the solid phase. More bitumen is then required to coat the mineral particles. If the amount of microsilica is too high, the effective binder film can decrease.

Similar non-linear effects have been reported in previous studies. For example, Mohammed and Ismael [48] tested 3, 6 and 9% silica fume by binder mass and found the best moisture-related performance at an intermediate dosage rather than at the highest content. Although their optimum dosage differs from that obtained in the present study (12%), the general existence of an intermediate optimum is consistent with the present results. Direct comparison of the numerical optimum is not appropriate because of differences in aggregate type, mixture composition and modification procedure.

### 3.2. Effect of Combined Use of Enriched Slag Aggregate and Microsilica-Modified Bitumen on the Mechanical Properties of Asphalt Concrete

**Figure 14:**
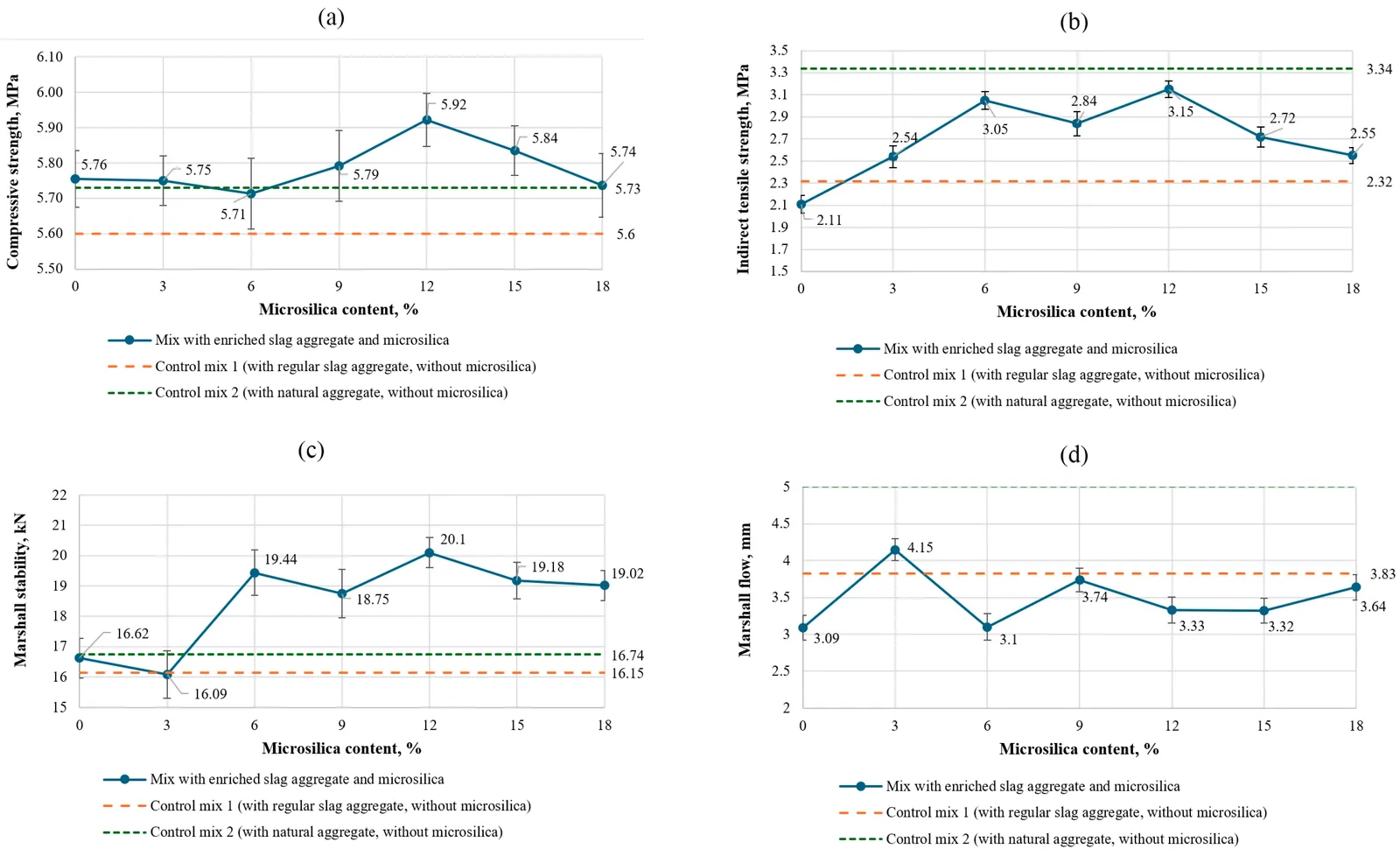
Effect of microsilica content on the mechanical properties of asphalt concrete with enriched slag aggregate compared with control mixtures: (**a**) compressive strength; (**b**) indirect tensile strength; (**c**) Marshall stability; (**d**) Marshall flow.

The results of evaluating the effects of slag aggregate enrichment and bitumen modification with microsilica on the mechanical properties (compressive strength, indirect tensile strength, Marshall stability, Marshall flow) of asphalt concrete are shown in Figure 14.

Compressive strength increased with microsilica content up to 12%, where the maximum value was 5.92 MPa. At 15 and 18%, the strength decreased. The 12% mixture showed higher compressive strength than the mixture with enriched slag aggregate without microsilica and both control mixtures. The higher strength of mixtures with enriched slag aggregate is consistent with the improved properties of the aggregate itself. Selective crushing removes weaker porous grains and retains denser particles with lower crushing value [22]. This produces a stronger coarse aggregate skeleton before any additional binder modification is applied.

The effect of microsilica was more pronounced for ITS. The value increased from about 2.11 MPa for the enriched-slag mixture without microsilica to 3.15 MPa at 12%. This corresponds to an increase of about 49%. The 12% mixture also showed substantially higher indirect tensile strength than the control mixture with regular slag aggregate. Also, the 12% mixture was only slightly inferior to the control mixture 2 with natural aggregate in terms of indirect tensile strength, with values of 3.15 and 3.24 MPa, respectively.

Similar trends have been reported for silica-based modifiers in the literature. Taherkhani and Tajdini [49] found that nano-silica increased indirect tensile strength and improved resistance to fatigue and freeze–thaw conditioning compared with hydrated lime. Tiwari et al. [47] reported that silica-fume-containing asphalt mixtures exhibited indirect tensile strength comparable to or slightly higher than that of mixtures containing conventional mineral filler.

The SEM results presented later in this section provide a possible explanation for the increase in indirect tensile strength. The modified mixture showed fewer large interfacial voids and a more continuous aggregate–mastic contact region. However, indirect tensile strength is a property of the whole asphalt concrete specimen and should not be interpreted as a direct measure of adhesion.

Marshall stability followed a similar trend. The maximum value of about 20.10 kN was obtained at 12% microsilica. At higher contents, stability decreased. Relative to control mixture 1 with regular slag aggregate, the increase at 12% was about 24.4%.

The mixture containing 12% microsilica also showed higher Marshall stability than control mixture 2 with natural aggregate. This difference may be partly attributed to the rougher and more angular surface of ACBFS particles. Such surface texture can increase mechanical interlocking with the bitumen–mineral mastic and enlarge the effective contact area between aggregate and binder. In the present study, this effect was combined with the use of enriched slag aggregate and microsilica-modified bitumen, which likely contributed to the higher resistance of the mixture to load-induced deformation.

Guo et al. [50] reported that, in their study, the Marshall stability of silane-silica-modified asphalt increased from 11.27 to 14.03 kN, corresponding to an improvement of about 24.4%. Residual stability after water immersion also increased. The numerical similarity should be treated only as a comparison of trends because that study used silane-treated silica and a different aggregate system.

The effect of slag aggregate physical and mechanical properties should also be considered. Previous studies on slag asphalt mixtures show that dense and mechanically stable slag aggregates can improve rutting and moisture resistance, while highly porous slag can increase binder absorption and reduce performance [51]. This is consistent with the use of enrichment in the present work to remove weak and highly porous particles before the aggregate is introduced into the asphalt mixture.

Marshall flow did not follow the same trend as stability. At 12% microsilica, the flow was about 3.33 mm while Marshall stability reached its maximum. This indicates that the increase in stability was not accompanied by a large increase in deformation.

The reduction in strength and stability at 15 and 18% microsilica indicates that excessive modifier content is not beneficial. At high contents, the increased surface area of the fine particles raises binder demand and can reduce the effective bitumen film thickness.

The combination of high Marshall stability (20.10 kN) and moderate flow (3.33 mm) at 12% microsilica suggests favorable resistance to load-induced deformation. This is consistent with the FHWA-RD-97-148 [52] guideline, which reports high stability, good flow characteristics, and satisfactory rutting resistance of asphalt mixtures containing ACBFS. However, Marshall stability and flow provide only indirect indications of deformation resistance and cannot substitute for direct rutting tests. Therefore, wheel-tracking and repeated-load tests, together with long-term durability assessments, are planned for subsequent research stages.

### 3.3. Effect of Combined Use of Enriched Slag Aggregate and Microsilica-Modified Bitumen on the Resistance of Asphalt Concrete to Climatic and Weather Factors

**Figure 15:**
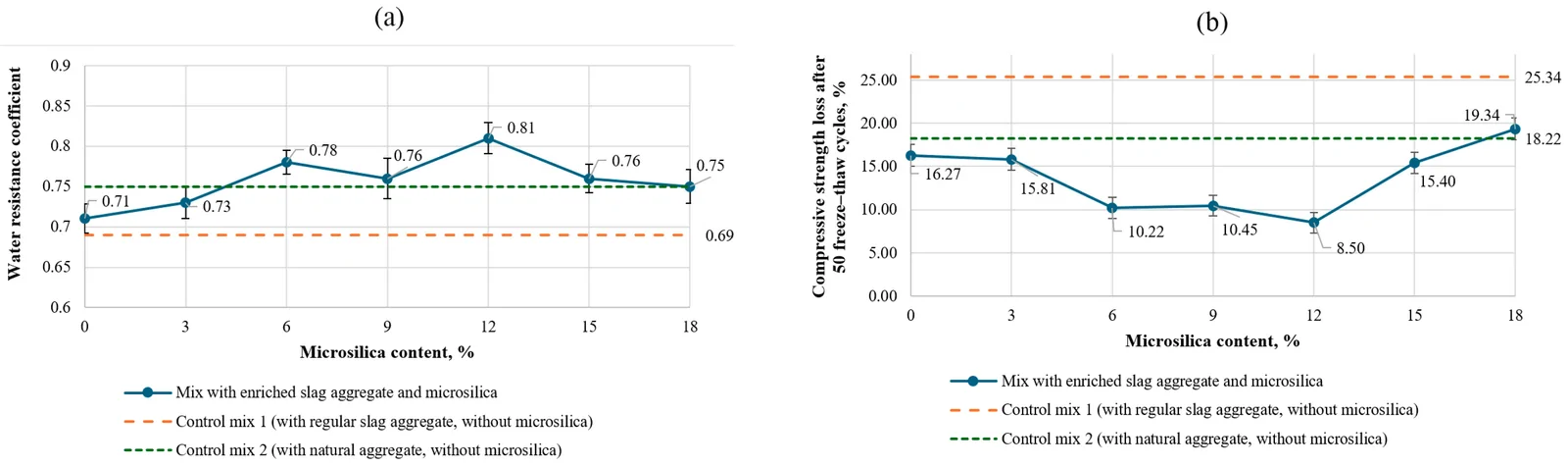
Effect of microsilica content on the resistance of asphalt concrete with enriched slag aggregate to climatic and weather factors compared with control mixtures: (**a**) water resistance coefficient; (**b**) compressive strength loss after 50 freeze–thaw cycles.

The results of evaluating the effects of slag aggregate enrichment and bitumen modification with microsilica on the resistance of asphalt concrete to climatic and weather factors (water resistance coefficient and compressive strength loss after 50 freeze–thaw cycles) are presented in Figure 15. Analysis of both properties shows that the combined modification is more effective than either the regular slag system or the enriched slag mixture without microsilica.

The water resistance coefficient increased with microsilica content and reached a maximum of about 0.81 at 12%. At 15 and 18%, the value decreased. The 12% mixture also exceeded both control mixtures.

The effect can be divided into two parts. Aggregate enrichment reduces the amount of porous slag particles that can absorb water. Previous work [22] on selectively crushed ACBFS showed a reduction in water absorption from 4.96 to 2.38% after enrichment. Other studies of slag enrichment also reported lower water absorption and higher freeze–thaw resistance after removal of weak porous grains [21].

The obtained results on the effect of microsilica-modified bitumen on the water resistance of asphalt concrete are in good agreement with the findings of Taher and Ismael [53]. They investigated hot-mix asphalt containing nano-silica added directly to the binder and reported lower moisture susceptibility together with higher tensile strength ratio values after modification. This supports the trend observed in the present study, where the water resistance coefficient increased with microsilica content up to 12% and then decreased at higher dosages. The improvement can be related to changes in the binder–mastic system, while the use of enriched slag aggregate additionally reduces the contribution of highly porous particles that can retain water.

Beyond the effects of aggregate porosity and binder modification, moisture resistance also depends on aggregate surface characteristics, mineral composition, and asphalt–aggregate adhesion. Recent studies provide further insight into these interfacial mechanisms. Xiao et al. [54] demonstrated that hydrophobic polyethylene coatings on acidic aggregates can modify surface energy, improve asphalt–aggregate adhesion, and reduce moisture-induced stripping. Zhou et al. [55] reported that mineral composition, surface polarity, and surface energy significantly influence interfacial adhesion, with carbonate minerals generally exhibiting greater adhesion stability than silicate minerals under moisture exposure. Zhu et al. [56] further showed that water molecules can form a hydration layer at the asphalt–aggregate interface, weakening bitumen adsorption and promoting stripping. These findings suggest that the improved moisture resistance observed in the present study may result from complementary effects at different structural levels. Selective crushing reduces the proportion of highly porous ACBFS particles and limits potential water accumulation, while microsilica modification may promote a more continuous aggregate–mastic contact region, as suggested by the SEM observations. The CaO- and SiO_2_-rich composition of ACBFS (Table 1) may also influence interfacial interactions, although the contributions of individual mineral phases were not directly investigated. Thus, the increase in the water resistance coefficient from approximately 0.69 to 0.81 can be associated with both reduced aggregate porosity and potentially improved interfacial characteristics. However, direct surface-energy and adhesion measurements are required to confirm these mechanisms. The combined reduction in water accumulation and improvement in aggregate–mastic contact may also contribute to the enhanced freeze–thaw resistance discussed below.

As shown by the analysis of the freeze–thaw test results, compressive strength loss decreased with increasing microsilica content up to 12%, reaching a minimum value of approximately 8.50% after 50 cycles. Compared with the mixture containing enriched slag aggregate without microsilica, the reduction was close to 48%. The compressive strength loss was also lower than for the control mixtures with regular slag aggregate and natural aggregate. At 15 and 18%, the compressive strength loss increased again.

The improvement in freeze–thaw resistance agrees with published results for silica-modified asphalt. Taherkhani and Tajdini reported that nano-silica improves indirect tensile strength after repeated freeze–thaw cycles and performed better than hydrated lime under the same conditioning [49].

The present results also show why enrichment of the slag aggregate is important. Porous ACBFS particles can retain water inside the grain. During freezing, this water can contribute to internal damage. Selective crushing removes part of these porous and weak grains before the asphalt mixture is produced [22]. The microsilica-modified binder then acts mainly at the mastic and aggregate–mastic contact level. The two modifications therefore affect different parts of the asphalt concrete structure.

This distinction is important because the improvement observed in the present study cannot be attributed to microsilica alone.

Overall, the results of Figure 13, Figure 14 and Figure 15 show that the best combination of physical, mechanical and durability properties was obtained at 12% microsilica by mass of bitumen. At this content, the mixture with enriched slag aggregate showed the highest or near-highest density, compressive strength, indirect tensile strength, Marshall stability and water resistance, together with the lowest strength loss after 50 freeze–thaw cycles. The improvement should be attributed to the combined effect of slag aggregate enrichment and bitumen modification with microsilica. Aggregate enrichment mainly reduces the proportion of weak and highly porous slag particles, whereas microsilica modification mainly affects the binder–mastic system. At microsilica contents above 12%, several properties deteriorated, indicating that the beneficial effect of the modifier is limited to a certain dosage range.

### 3.4. Microstructural and Elemental Analysis of Asphalt Concrete with Enriched Slag Aggregate and Microsilica-Modified Bitumen

To explain the changes observed in the physical, mechanical, moisture and freeze–thaw properties, microstructural and elemental analyses were carried out for two representative mixtures: the control asphalt concrete with regular slag aggregate and unmodified bitumen, and the mixture with enriched slag aggregate and 12% microsilica-modified bitumen. The latter composition was selected because it showed the best overall combination of the investigated properties, including the highest or near-highest strength and water resistance together with the lowest strength loss after 50 freeze–thaw cycles. The control mixture was selected as the reference state because it contained regular, non-enriched slag aggregate and no microsilica. The intermediate microsilica contents were not examined by SEM and EDS because the purpose of the microstructural study was not to trace the gradual effect of microsilica dosage, but to compare the initial slag asphalt concrete with the composition that showed the most pronounced overall improvement after combined aggregate enrichment and binder modification.

**Figure 16:**
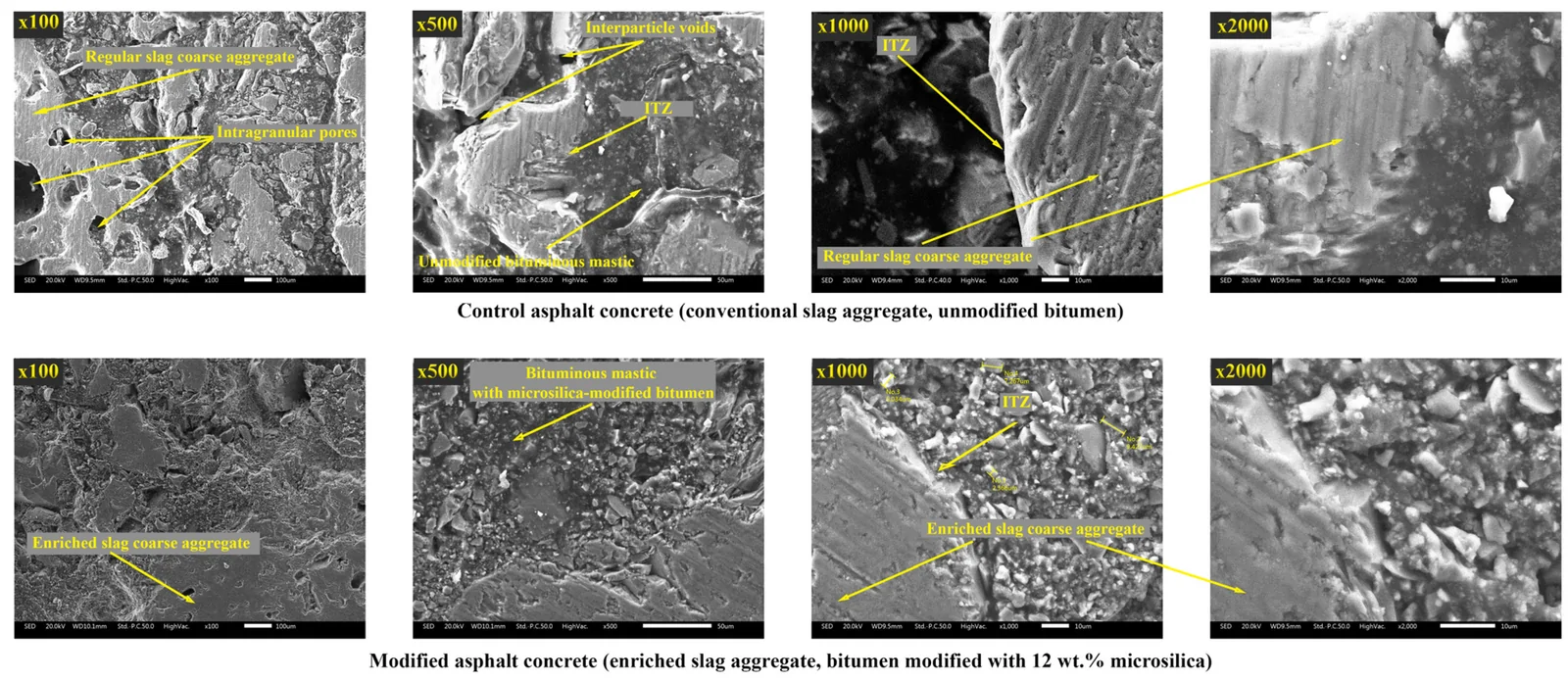
SEM micrographs of the control asphalt concrete (**top** row) and modified asphalt concrete (**bottom** row) at different magnifications.

SEM micrographs of the control and selected modified mixtures are presented in Figure 16. Comparative analysis revealed differences in both the morphology of the slag aggregate and the nature of its contact with the bitumen–mineral mastic. The most pronounced differences were observed in the number and size of structural voids, the surface condition of the slag particles, and the morphology of the slag aggregate–bitumen mastic interfacial region.

The micrograph of the control specimen shows a pronounced structural heterogeneity. In particular, a considerable number of relatively large internal pores are visible within individual slag particles, which is characteristic of ACBFS that has not undergone preliminary enrichment. In addition, interparticle and interfacial voids are observed between the mineral particles and in the immediate vicinity of the slag–bitumen mastic interface (Figure 16).

The microscopic morphology of ACBFS provides further insight into the structural differences between the investigated asphalt concrete mixtures. As illustrated by the SEM micrograph of the original ACBFS aggregate (Figure 1b) and the micrographs of the control asphalt concrete (Figure 16), regular slag particles exhibit a heterogeneous structure with irregular surfaces and pores of different sizes. Some pores are located within individual slag grains (intragranular pores), whereas additional voids occur between neighboring particles and in the aggregate–mastic interfacial transition zone (ITZ). These morphological features may facilitate water penetration and contribute to local stress concentrations within the asphalt concrete structure.

In contrast, SEM observations of the mixture containing enriched ACBFS and 12% microsilica-modified bitumen (Figure 16) reveal fewer large pores within the observed slag particles and a more continuous contact region between the aggregate and the surrounding bitumen–mineral mastic. The reduced occurrence of intragranular pores is primarily associated with the selective removal of weak, highly porous particles during aggregate enrichment, rather than with a reduction in the porosity of individual grains caused by microsilica modification. These observations support the role of aggregate enrichment in improving the structural quality of the mineral skeleton of asphalt concrete.

The presence of large internal pores in the control aggregate particles should be attributed primarily to the structure of the original regular slag aggregate. Such defects are formed during the generation and cooling of blast-furnace slag and remain after crushing. Therefore, the reduced occurrence of these pores in the modified mixture (Figure 16) should be considered mainly as a result of using enriched slag aggregate rather than as a direct effect of microsilica addition.

Particular attention should be paid to the nature of the interface between the slag aggregate and the surrounding bitumen–mineral mastic. In the control specimen, this interface is relatively sharp in some areas (Figure 16). Local unfilled regions and interfacial voids are observed between the mineral particle surface and the mastic. Such morphology indicates incomplete contact between the components and may be regarded as a sign of less effective interfacial interaction.

The microstructure of the modified specimen (Figure 16) is characterized by a denser structure of the contact region. A well-developed, finely dispersed bitumen–mineral mastic is observed on the surface of the slag aggregate, showing more continuous contact with the irregular mineral surface. The SEM micrographs of the modified specimen reveal considerably fewer large unfilled interfacial gaps. Within the examined area, the modified specimen appears more compact, and the boundary between the aggregate and the surrounding mastic is less pronounced.

This observation is consistent with the expected physical effect of microsilica addition. Owing to their high fineness, microsilica particles become incorporated into the bitumen–mineral mastic and can fill small interparticle spaces and surface irregularities of the aggregate.

**Figure 17:**
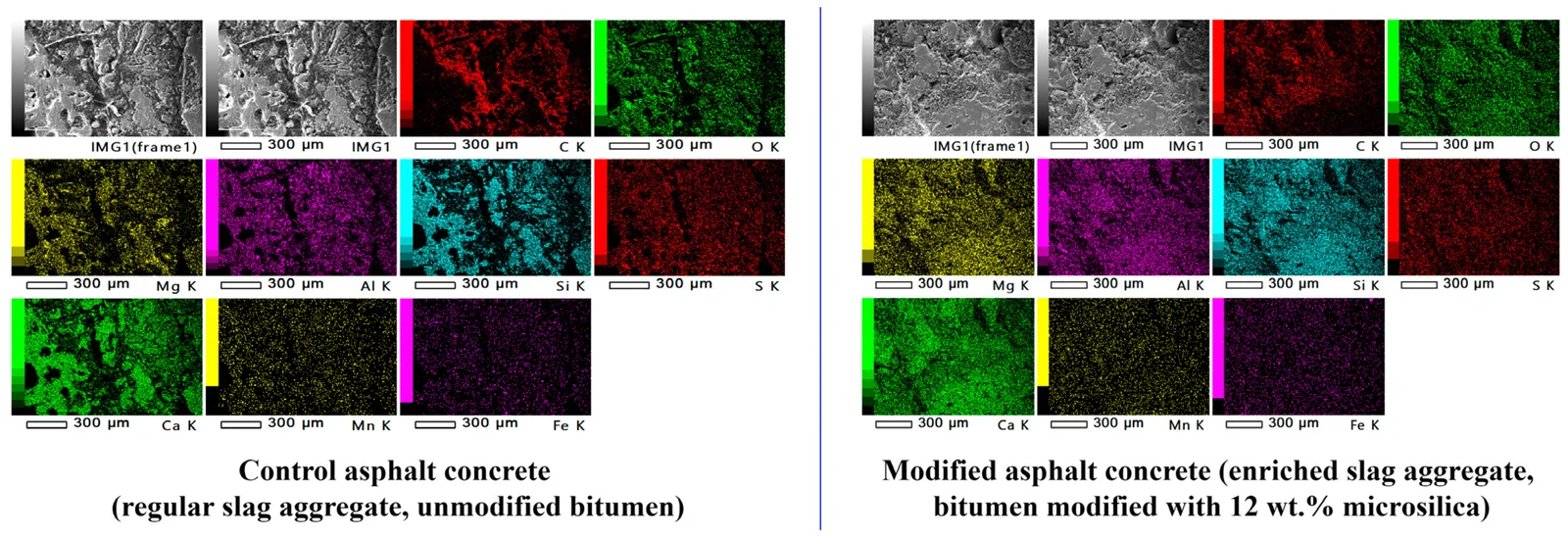
Comparison of EDS maps of the control and modified mixtures at the same magnification (×100). The colors indicate the elemental distribution: red—carbon (C) and sulfur (S); green—oxygen (O) and calcium (Ca); yellow—magnesium (Mg); purple—aluminum (Al) and iron (Fe); cyan—silicon (Si).

Thus, SEM analysis made it possible to identify two different mechanisms contributing to the improved structure of the modified asphalt concrete specimen. The first is associated with the use of enriched slag aggregate instead of regular slag aggregate and is reflected in a more favorable morphology of the coarse aggregate itself, including a reduced occurrence of pronounced porosity in individual particles. The second is likely related to the addition of microsilica and is manifested mainly in the densification of the bitumen–mineral mastic, a reduction in unfilled interparticle and interfacial spaces, and the formation of a more continuous contact region between the mastic and the slag surface. The observed changes in the homogeneity of the system are indirectly supported by the EDS mapping results presented in Figure 17.

Comparison of the Si, Ca, and Al maps shows that the control specimen is characterized by a more pronounced alternation of regions with different signal intensities. In the modified mixture, the Si distribution becomes more continuous and less localized, while the contrast between individual Ca- and Al-containing regions is reduced.

The reduced variability of Si has the most direct physical explanation. Microsilica consists of highly dispersed SiO_2_ and, after mixing, becomes incorporated into the bitumen–mineral mastic. Therefore, the increase in the average Si content together with the simultaneous reduction in its variation between local fields is consistent with a more uniform dispersion of the silica-containing additive throughout the mastic.

The more uniform distribution of Ca and Al should not be interpreted as a direct “redistribution” of these elements by microsilica. Microsilica is not a significant source of either Ca or Al. The observed reduction in their inter-field variability is primarily related to differences in the mineral aggregate itself, since the mixture containing enriched slag aggregate is characterized by a different morphology. This may reduce the contribution of large, chemically contrasting Ca- and Al-rich regions. A more finely dispersed bitumen–mineral mastic containing uniformly distributed microsilica may also contribute to the visually more homogeneous EDS maps by reducing the sharpness of transitions between individual mineral regions.

**Figure 18:**
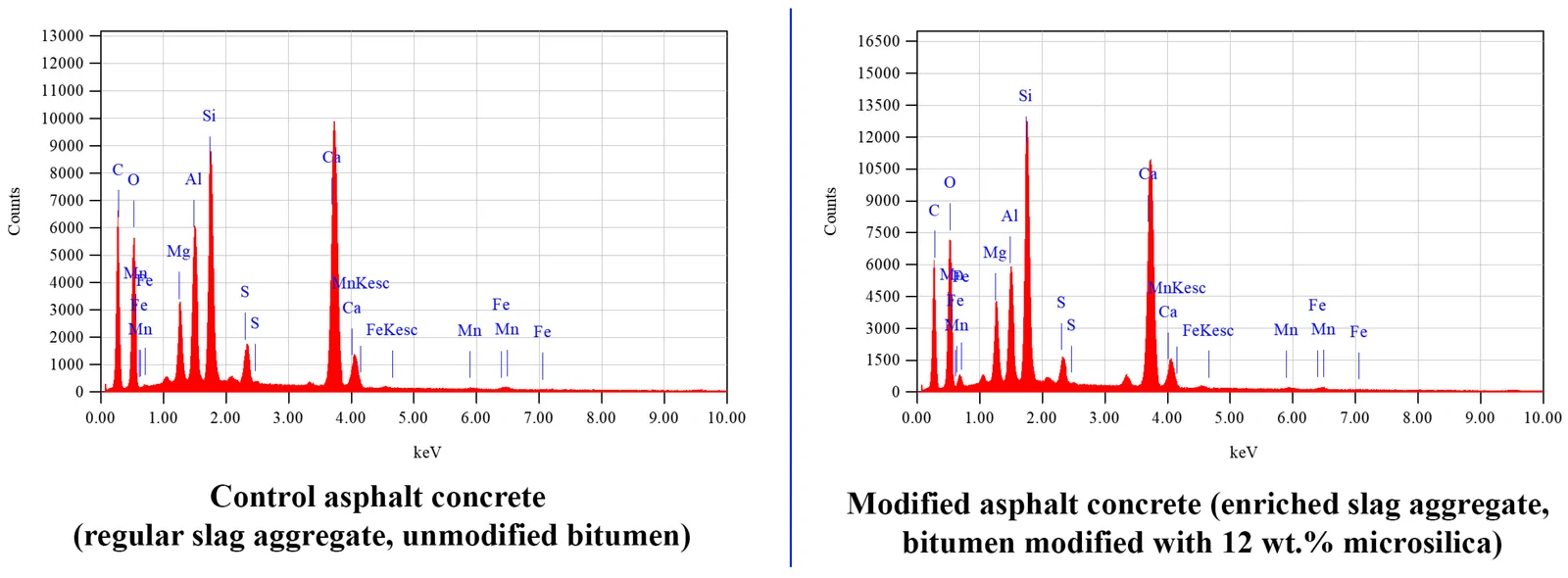
Comparison of representative EDS spectra of the control and modified mixtures at the same magnification (×1000).

To confirm the qualitative elemental composition identified by EDS mapping, Figure 18 presents representative EDS spectra of the control and modified specimens obtained at the same magnification (×1000). For each composition, the spectrum of the field whose Mg, Al, Si, and Ca contents were closest to the group mean was selected. This approach provides a characteristic spectral profile without substituting a single local spectrum for the statistical analysis of all examined fields.

The main peaks of C, O, Mg, Al, Si, S, and Ca are detected in both spectra, together with trace signals of Mn and Fe. In the selected field of the control specimen, the contents of Si, Ca, Al, and Mg were 14.85, 21.53, 9.83, and 4.83 wt.%, respectively. In the representative field of the modified specimen, the corresponding values were 18.80, 22.47, 8.48, and 5.59 wt.%. The higher Si content in the modified mixture is consistent with the addition of microsilica.

Thus, the modified specimen exhibits a more spatially continuous distribution of Si, which is consistent with the incorporation of highly dispersed microsilica into the bituminous binder. Together with the SEM observations of a denser and more continuous contact region, this indicates the formation of a more homogeneous bitumen–mineral mastic around the slag aggregate and indirectly suggests improved interaction between the modified bituminous binder and the particles of the preliminarily enriched slag aggregate.

**Figure 19:**
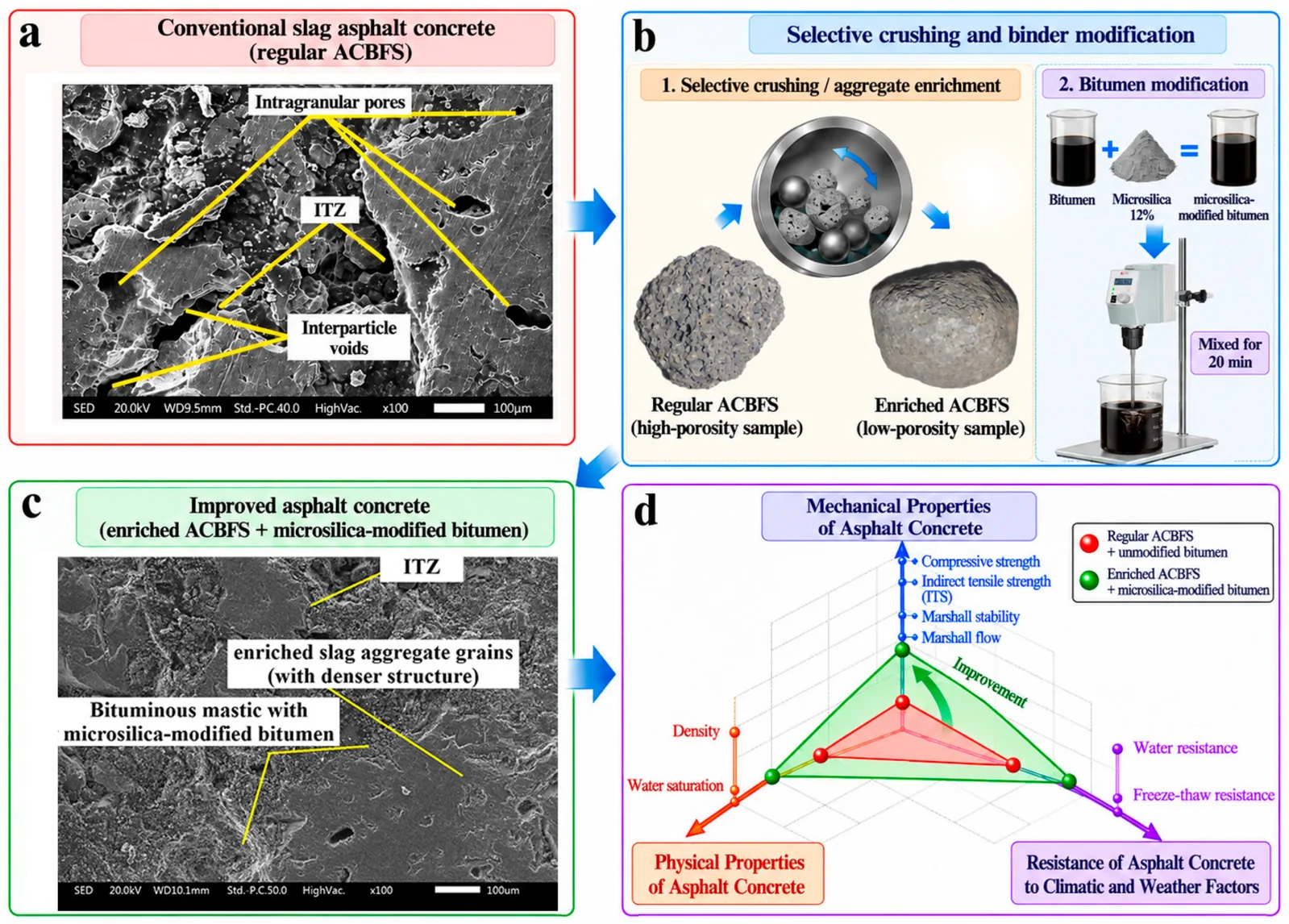
Schematic illustration of the proposed microstructural mechanisms and associated improvements in ACBFS-based asphalt concrete: (**a**) SEM micrograph of the control asphalt concrete containing regular ACBFS and unmodified bitumen; (**b**) aggregate enrichment by selective crushing and preparation of bitumen modified with 12% microsilica; (**c**) SEM micrograph of the modified asphalt concrete containing enriched ACBFS and microsilica-modified bitumen; (**d**) qualitative schematic representation of the improvements in physical, mechanical, and moisture- and freeze–thaw-related performance characteristics.

The proposed microstructural mechanisms underlying the improvement of ACBFS-based asphalt concrete are schematically illustrated in Figure 19. Two complementary modification mechanisms can be distinguished, affecting different structural constituents of the material.

The first mechanism is associated with the preliminary enrichment of ACBFS aggregate by selective crushing (Figure 19b). During this process, weaker and more porous slag particles are preferentially fragmented, while denser and mechanically resistant grains retain their integrity. Subsequent separation of the fragmented material increases the proportion of low-porosity particles in the enriched aggregate. This mechanism is consistent with the SEM observations, which reveal numerous intragranular pores in the regular slag particles (Figure 19a) and fewer large pores in the enriched aggregate incorporated into the modified asphalt concrete (Figure 19c). Therefore, selective crushing improves the quality of the aggregate primarily by removing weak and highly porous grains rather than by directly reducing the internal porosity of individual particles.

The second mechanism is associated with the modification of the bituminous binder with microsilica (Figure 19b). The incorporation of finely dispersed microsilica into bitumen influences the structure of the binder–mineral mastic system. SEM observations of the modified asphalt concrete reveal a well-developed, finely dispersed bitumen–mineral mastic and more continuous contact with the irregular surfaces of the enriched slag particles (Figure 19c). Compared with the control mixture, fewer large unfilled regions are observed in the aggregate–mastic interfacial transition zone. These findings, together with the EDS observations of a more continuous Si distribution, are consistent with improved structural continuity of the modified mastic and its contact with the mineral aggregate.

The structural changes associated with these two modification mechanisms may contribute to the improvements in the physical, mechanical, and durability characteristics of asphalt concrete, as qualitatively illustrated in Figure 19d. Aggregate enrichment reduces the contribution of weak, highly porous particles to the mineral skeleton, while microsilica modification is associated with improved continuity of the binder–mastic system and the aggregate–mastic contact region. These changes are consistent with the experimentally observed improvements in density, compressive strength, indirect tensile strength, Marshall stability, water resistance, and resistance to repeated freeze–thaw cycles, together with reduced water saturation. Thus, the combined application of aggregate enrichment and microsilica modification of bitumen provides a comprehensive approach to improving the microstructure of ACBFS-based asphalt concrete, contributing to enhanced mechanical performance, moisture resistance, and freeze–thaw durability.

## 4. Conclusions

The present study evaluated the combined effect of selective enrichment of ACBFS aggregate and modification of bitumen with microsilica. The asphalt concrete containing regular ACBFS and unmodified bitumen was used as the main control mixture. Based on the obtained results, the following conclusions can be drawn:

1. Selective crushing improved the quality of ACBFS coarse aggregate by preferentially removing weak and highly porous particles. Water absorption decreased from 4.98 to 2.38%, abrasion loss from 45.54 to 32.05%, and aggregate crushing value from 30.82 to 18.4%.

2. The combined use of enriched ACBFS coarse aggregate and microsilica-modified bitumen improved the physical and mechanical properties of asphalt concrete. At 12% microsilica by bitumen mass, compressive strength reached 5.92 MPa, indirect tensile strength 3.15 MPa, and Marshall stability 20.10 kN, representing improvements of approximately 6%, 12%, and 24.4%, respectively, compared with the regular-slag control mixture.

3. Moisture and freeze–thaw resistance also improved substantially. At 12% microsilica, the water resistance coefficient increased from approximately 0.69 to 0.81, while compressive strength loss after 50 freeze–thaw cycles decreased from 23.94 to 8.50%. Higher microsilica contents (15–18%) reduced several performance characteristics, indicating that 12% was the most effective dosage within the investigated range.

4. SEM–EDS analysis revealed fewer large interfacial voids, a more continuous aggregate–mastic contact region, and a more uniform Si distribution in the modified mixture. These observations support the complementary roles of aggregate enrichment and binder modification in improving asphalt concrete performance.

5. Despite the remaining differences between enriched ACBFS and natural aggregate, the developed asphalt concrete shows potential for applications on low-volume roads. However, the identified 12% microsilica dosage is specific to the investigated materials and mixture design, while direct rutting resistance, long-term durability testing, and field validation remain necessary before practical implementation.

## Figures and Tables

**Table 1:** Chemical composition of ACBFS [21].

**Chemical Compound**	CaO	SiO_2_	Al_2_O_3_	MgO	MnO	FeO	S	TiO_2_
**Mass fraction, %**	44.3	40.7	8.0	5.2	0.2	0.5	0.65	0.45

**Table 2:** Physical and mechanical properties of the aggregates.

Property	Regular Slag Aggregate	Enriched Slag Aggregate	Natural Aggregate	Standard Used for Testing
Bulk density (kg/m^3^)	1197	1390	1450	GOST 32822-2014 [29]
Water absorption (%)	4.98	2.38	0.52	GOST 32815-2014 [30] (comparable in scope to ASTM C 127 [31])
Content of flaky and elongated aggregate grains (%)	12.42	0.24	4.85	GOST 32864-2014 [32] (comparable in scope to ASTM D 4791 [33])
Loss of the sample mass after abrasion test (%)	45.54	32.05	11.15	GOST 8269.0-97 [34] (comparable in scope to ASTM C131 [35])
Aggregate crushing value (%)	30.82	18.4	5.6	GOST 32817-2014 [36] (comparable in scope to BS 812-110:1990 [37])

**Table 3:** Characteristics of the bituminous binder.

Property	Value
Bitumen grade (producer)	BND 100/130 (JSC KazBitumService)
Ring-and-ball softening point, °C	46
Penetration at 25 °C, 0.1 mm	112
Ductility at 25 °C, cm	115
Mass change after heating, %	0.1

**Table 4:** Chemical composition of microsilica.

**Chemical Compound**	SiO_2_	Al	CaO	MgO	Cr	Mn	C	S
**Mass fraction, %**	94.37	0.69	0.85	1.01	0.61	1.23	0.98	0.26

## Data Availability

The original contributions presented in this study are included in the article. Further inquiries can be directed to the corresponding author.

## References

[1] Chen L., Yang M., Chen Z., Xie Z., Huang L., Osman A.I., Farghali M., Sandanayake M., Liu E., Ahn Y.H. (2024). Conversion of Waste into Sustainable Construction Materials: A Review of Recent Developments and Prospects. Mater. Today Sustain..

[2] Ke Y., Chen Y., Liang S., Hu J., Hou H., Quan J., Li X., Duan H., Yuan S., Yang J. (2023). Environmentally Sound Management of Industrial Solid Waste: A Paradigm of Proposed Bi-Tetrahedron. Resour. Conserv. Recycl..

[3] Ma D., Gao X., Zhang J. (2025). Co-Exploitation of Mine-Derived Geothermal Energy: Recent Advances and Emerging Perspectives. GeoEnergy Commun..

[4] Ma G., Ma D., Gao X., Wang L., Tang Z. (2026). Effect of Relative Humidity on the Fracture and Creep Behaviors of Cement Paste: Insights from Micro-Indentation and ^1^H NMR Testing. Constr. Build. Mater..

[5] Wang F., Sun W., Long G., Wang Y., Hu Q., Shangguan M., He J., Zhou X., Ma G., Zhou J.L. (2026). Interfacial-Engineered Pb^2+^ Sequestration in Calcium Sulfoaluminate Hybrid Binders: Coupled Precipitation–Ettringite Uptake for Transport Regulation and Strength Gain. Adv. Compos. Hybrid Mater..

[6] Jahangirnejad S., Van Dam T., Morian D., Smith K., Perera R., Tyson S. (2013). Blast Furnace Slag as Sustainable Material in Concrete Pavements. Transp. Res. Rec..

[7] An J., Song Y., Wu S., Xu H., Yang X., Gong Z., Hao H. (2026). Investigation on the Moisture Resistance and Mechanism of Asphalt Concrete Using Metallurgical Solid Waste as Anti-Stripping Agent. Constr. Build. Mater..

[8] Mohamed O.A., Ghanam O., Hamdan A., Zuaiter M., Kim T.-Y. (2025). Mechanical Properties and Durability of Concrete with Recycled Air-Cooled Blast Furnace Slag Aggregates. Sci. Rep..

[9] Cao Q., Nawaz U., Jiang X., Zhang L., Ansari W.S. (2022). Effect of Air-Cooled Blast Furnace Slag Aggregate on Mechanical Properties of Ultra-High-Performance Concrete. Case Stud. Constr. Mater..

[10] Rondón-Quintana H.A., Ruge-Cárdenas J.C., de Farias M.M. (2019). Behavior of Hot-Mix Asphalt Containing Blast Furnace Slag as Aggregate: Evaluation by Mass and Volume Substitution. J. Mater. Civ. Eng..

[11] Rondón H.A., Chaves S., Escobar D. (2018). Evaluation of a Warm Mix Asphalt Manufactured with Blast Furnace Slag. Mod. Appl. Sci..

[12] Stępień J., Maciejewski K., Ramiączek P., Chomicz-Kowalska A. (2026). Effect of Steel Slag and Air-Cooled Blast Furnace Slag Aggregates on the Performance of Warm-Mix Asphalt Concrete Produced with Foamed Bitumen. Materials.

[13] Moura B.L.R., Teixeira J.E.S.L., Simão R.A., Khedmati M., Kim Y.-R., Pires P.J.M. (2020). Adhesion between Steel Slag Aggregates and Bituminous Binder Based on Surface Characteristics and Mixture Moisture Resistance. Constr. Build. Mater..

[14] Kunaev V.A., Akhmetova G.E., Ospanova A.Z., Kenzhaliyev O.B., Tavshanov I.S., Fathi M.S., Ulyeva G.A., Batyrbek A.E., Charnyi D.Y. (2026). Blast Furnace Slag as an Alternative to Natural Mineral Materials in Pavement Engineering: A Review. Kompleks. Ispolz. Miner. Syra.

[15] Kadyrov A.S., Kunaev V.A., Georgiadi I.V. (2017). Ferrous Metallurgy Waste and Waste Technical Fluids for Obtaining the Material of Road Bases. Ecol. Ind. Russ..

[16] Georgiadi I., Kadyrov A., Kunaev V. (2023). Development of the Universal Working Equipment of the Excavator for Preparation of Materials Based on Waste during the Road Building. Commun.-Sci. Lett. Univ. Zilina.

[17] Shatnawi A.S. (2008). Effect of Jordanian Steel Blast Furnace Slag on Asphalt Concrete Hot Mixes. Jordan J. Civ. Eng..

[18] Kunaev V., Tavshanov I., Asanov R. (2024). Laboratory Evaluation of a Complex Treatment Technology for Reducing Water Absorption of the Pavement Subbase Aggregate from the Blast-Furnace Slag. Eng. Res. Express.

[19] Zou G., Sun X., Liu X., Zhang J. (2020). Influence Factors on Using Recycled Concrete Aggregate in Foamed Asphalt Mixtures Based on Tensile Strength and Moisture Resistance. Constr. Build. Mater..

[20] Al-Busaltan S., Al Nageim H., Atherton W., Sharples G. (2022). Performance of Porous Asphalt Mixtures Containing Recycled Concrete Aggregate and Fly Ash. Materials.

[21] Kunaev V., Bazarov B., Kadyrov A., Konakbaeva A. (2024). Selective Crushing, Enrichment by Friction Properties and Hydrophobization for Obtaining the Sustainable Blast Furnace Slag Aggregate for Road Subbase. Ain Shams Eng. J..

[22] Kunaev V., Akhmetova G., Zhautikov B., Batyrbek A., Tavshanov I., Charnyi D., Kydyrbayeva S., Kamarova S., Fathi M.S., Suleyev B. (2025). Enhancing Physical-Mechanical Properties of Coarse Slag Aggregate via Selective Crushing for Asphalt Concrete. Results Eng..

[23] Yusoff N.I.M., Breem A.A.S., Alattug H.N.M., Hamim A., Ahmad J. (2014). The Effects of Moisture Susceptibility and Ageing Conditions on Nano-Silica/Polymer-Modified Asphalt Mixtures. Constr. Build. Mater..

[24] Long Z., You L., Tang X., Ma W., Ding Y., Xu F. (2020). Analysis of Interfacial Adhesion Properties of Nano-Silica Modified Asphalt Mixtures Using Molecular Dynamics Simulation. Constr. Build. Mater..

[25] Sezavar R., Shafabakhsh G., Mirabdolazimi S.M. (2019). New Model of Moisture Susceptibility of Nano Silica-Modified Asphalt Concrete Using GMDH Algorithm. Constr. Build. Mater..

[26] Masri K.A., Awang H., Jaya R.P., Ali M.I., Ramli N.I., Arshad A.K. (2019). Moisture Susceptibility of Porous Asphalt Mixture with Nano Silica Modified Asphalt Binder. IOP Conf. Ser. Earth Environ. Sci..

[27] Chen L., Gu W., Zhang X. (2021). Environment Effect on the Rutting Resistance of Nano-SiO_2_-Modified Asphalt Concrete: Temperature and Water. Adv. Civ. Eng..

[28] Al-Zarjawi A.K., Mahdi Z.A., Abbas H.H. (2020). Effect of Silica Fume on Cold Mix Asphalt Mixture. IOP Conf. Ser. Mater. Sci. Eng..

[29] (2019). Public Roads. Crushed Stone and Gravel from Rocks—Determination of Bulk Density and Voidness.

[30] (2015). Public Roads. Slag Aggregate—Determination of Average Density and Water Absorption.

[31] (2012). Standard Test Method for Density, Relative Density (Specific Gravity), and Absorption of Coarse Aggregate.

[32] (2015). Public Roads—Determination of the Content of Flaky and Elongated Aggregate Grains.

[33] (2019). Standard Test Method for Flat Particles, Elongated Particles, or Flat and Elongated Particles in Coarse Aggregate.

[34] (2018). Crushed Stone and Gravel from Dense Rocks and Industrial Waste for Construction Works—Methods of Physical and Mechanical Tests.

[35] (2020). Standard Test Method for Resistance to Degradation of Small-Size Coarse Aggregate by Abrasion and Impact in the Los Angeles Machine.

[36] (2015). Public Roads. Slag Aggregate—Determination of Crushability.

[37] (1990). Testing Aggregates—Part 110: Methods for Determination of Aggregate Crushing Value (ACV).

[38] Abishkenov M., Tavshanov I., Lutchenko N., Nogayev K., Ashkeyev Z., Kulidan S. (2025). Hybrid Al6060/TiB_2_/Microsilica Composites Produced by Ultrasonically Assisted Stir Casting and Radial-Shear Rolling: Microstructural Evolution and Strength–Ductility Balance. Eng.

[39] (1998). Materials Based on Organic Binders for Road and Airfield Construction—Test Methods.

[40] (2005). Slag Crushed Stone and Sand for Road Construction—Technical Specifications.

[41] (2015). Public Roads—Slag Crushed Stone and Sand—Technical Requirements.

[42] (2019). Public Roads—Road Asphalt Mixtures and Asphalt Concrete—Volumetric-Functional Design System—Technical Requirements.

[43] (2022). Aggregates for Asphalt.

[44] Indiana Department of Transportation (2013). Inspection and Sampling Procedures for Fine and Coarse Aggregates.

[45] Highways England (2021). Manual of Contract Documents for Highway Works; Volume 1, Specification for Highway Works.

[46] (2002). Aggregates for Bituminous Mixtures and Surface Treatments for Roads, Airfields and Other Trafficked Areas.

[47] Tiwari N., Rondinella F., Satyam N., Baldo N. (2023). Experimental and machine learning approach to investigate the mechanical performance of asphalt mixtures with silica fume filler. Appl. Sci..

[48] Mohammed W.J., Ismael M.Q. (2025). Investigating the moisture susceptibility resistance of asphalt mixes modified with silica fume additive. J. Eng..

[49] Taherkhani H., Tajdini M. (2019). Comparing the effects of nano-silica and hydrated lime on the properties of asphalt concrete. Constr. Build. Mater..

[50] Guo X., Sun M., Dai W., Chen S. (2016). Performance Characteristics of Silane Silica Modified Asphalt. Adv. Mater. Sci. Eng..

[51] Skaf M., Manso J.M., Aragón Á., Fuente-Alonso J.A., Ortega-López V. (2017). EAF Slag in Asphalt Mixes: A Brief Review of Its Possible Re-Use. Resour. Conserv. Recycl..

[52] Federal Highway Administration (1997). Blast Furnace Slag—User Guideline: Asphalt Concrete. User Guidelines for Waste and Byproduct Materials in Pavement Construction.

[53] Taher Z.K., Ismael M.Q. (2023). Moisture Susceptibility of Hot Mix Asphalt Mixtures Modified by Nano Silica and Subjected to Aging Process. J. Eng..

[54] Xiao R., Zhang M., Zhong J., Baumgardner G.L., Huang B. (2025). Waste Plastic Powder Coating on Acidic Aggregates: A New Hydrophobic Coating Technology to Build Moisture-Resistant Asphalt Mixtures. Transp. Res. Rec..

[55] Zhou T., Wan S., Dong Z., Xie S., You L., Jin C. (2026). Physicochemical Mechanisms of Asphalt–Aggregate Interface Adhesion: Effects of Mineral Composition and Surface Energy. Constr. Build. Mater..

[56] Zhu S., Peng Y., Kong L., Wang D., Zhu H., Kane M., Ren Z., Li Z. (2025). Multi-Scale Method for Investigating the Mechanism of Water Effect on Asphalt/Aggregate Interface Adhesion and the Enhancement Role of Oxides. Energy.

